# Aerospace Bionic Robotics: BEAM-D Technical Standard of Biomimetic Engineering Design Methodology Applied to Mechatronics Systems

**DOI:** 10.3390/biomimetics10100668

**Published:** 2025-10-05

**Authors:** Jose Cornejo, Alfredo Weitzenfeld, José Baca, Cecilia E. García Cena

**Affiliations:** 1 Escuela Técnica Superior de Ingeniería y Diseño Industrial, Universidad Politécnica de Madrid, Ronda de Valencia, 3, 28012 Madrid, Spain; jose.baca@tamucc.edu (J.B.); cecilia.garcia@upm.es (C.E.G.C.); 2 Biorobotics Laboratory, Department of Computer Science and Engineering, University of South Florida, Tampa, FL 33620, USA; aweitzenfeld@usf.edu; 3 Department of Engineering, College of Engineering and Computer Science, Texas A&M University-Corpus Christi, Corpus Christi, TX 78412, USA; 4 Centre for Automation and Robotics (UPM-CSIC), Ronda de Valencia, 3, 28012 Madrid, Spain

**Keywords:** space exploration, orbital robotics, planetary robotics, biomimetics design, mechatronics design

## Abstract

The origin of life initiated an evolutionary continuum yielding biologically optimized systems capable of operating under extreme environmental constraints. Biomimetics, defined as the systematic abstraction and transfer of biological principles into engineering domains, has become a strategic design paradigm for addressing the multifactorial challenges of space systems. This study introduces two core contributions to formally establish the discipline of Aerospace Bionic Robotics (ABR): First, it elucidates the relevance of biologically derived functionalities such as autonomy, adaptability, and multifunctionality to enhance the efficiency of space robotic platforms operating in microgravity environments. Second, it proposed the BEAM-D (*Biomimetic Engineering and Aerospace Mechatronics Design*), a standard for the development of Aerospace Bionic Robotics. By integrating biological abstraction levels (morphological, functional, and behavioral) with engineering protocols including ISO, VDI, and NASA’s TRL, BEAM-D enables a structured design pathway encompassing subsystem specification, cyber–physical integration, in situ testing, and full-scale mission deployment. It is implemented through a modular BEAM-DX framework and reinforced by iterative BIOX design steps. This study thus establishes formalized bio-inspired design tools for advanced orbital and planetary robotic systems capable of sustained autonomous operations in deep space exploration scenarios.

## 1. Introduction

A billion years ago, life first emerged upon the Earth, inaugurating an evolutionary odyssey defined by continuous adaptation [1]. Through an unceasing process of trial and error, nature has sculpted an immense diversity of life forms, each exquisitely attuned to the challenges of its environment [2]. This heritage constitutes a vast and time-honored compendium of functional strategies, efficient and sustainable by design, offering a profound source of inspiration for contemporary science and technology [3]. Following this context, bio-inspiration emerges as a creative impulse rooted in the attentive observation of biological systems, catalyzing novel design ideas [4]. Expanding upon this, biomimetics, also known as biomimicry, was first formally defined by Janine Benyus in 1997 as ‘innovation inspired by nature’ [5]. The term originates from the Greek words bios (life) and mimesis (imitation). This approach advocates for innovation through a deep engagement with nature, viewing it as: (a) a model to emulate, (b) a measure for evaluating sustainability and performance, and (c) a mentor offering time-tested wisdom for solving complex challenges [6]. It advances this dialogue further, establishing a structured collaboration between biology, technology, and allied fields of innovation [7]. Through the analytical study of biological functions, their abstraction into transferable models, and their application to real-world problems, biomimetics becomes both a method and a bridge between the living world and engineered solutions [8,9]. In parallel, more technically inclined bionics focuses on enhancing or replacing biological functions using electronic or mechanical systems, offering a pathway for translating life’s intricacies into high-performance technological artifacts [10]. These fields are not acts of imitation, but rather of abstraction and translation, distilling biological intelligence into applicable technological models. The methodical observation of living-neuroethology systems [11], coupled with the extraction of fundamental principles to guide the development of biomimetic systems [12,13], has become a cornerstone of space engineering. The convergence of artificial vision and computational modeling now enables researchers to examine the dynamic behaviors with unprecedented precision [14].

To support this rapidly advancing field, the International Organization for Standardization (ISO) established the Technical Committee 266 [15]. It is tasked with developing standards specific to biomimetics, ensuring terminological clarity and reinforcing the scientific, commercial, and legal validity of methods and products. Though adoption of these standards remains voluntary, their invocation in legislation or contractual agreements confers legal enforceability, thereby enhancing regulatory trust, liability frameworks, and market readiness. With over 23,000 standards in place across all industrial sectors, ISO plays a pivotal role in shaping global innovation [9,16]. In addition, Stone et al., underscore the essence of bio-inspired design as the systematic extraction of biological knowledge to resolve specific engineering challenges [5,17]. The influence of bio-inspired engineering is already evident across a spectrum of industries [18,19]. In aerospace, the lightweight yet mechanically robust architecture of avian skeletons has inspired novel materials; in hydrodynamics, the morphology of marine life has guided the design of streamlined vehicles and fluid systems [20]. These innovations exemplify nature’s capacity to inform high-performance systems that simultaneously reduce energy consumption and mitigate environmental impact [21]. Mechanical engineering, in particular, has embraced these insights to develop devices and processes that are not only more efficient but also inherently more adaptable and durable [22,23].

**Figure 1:**
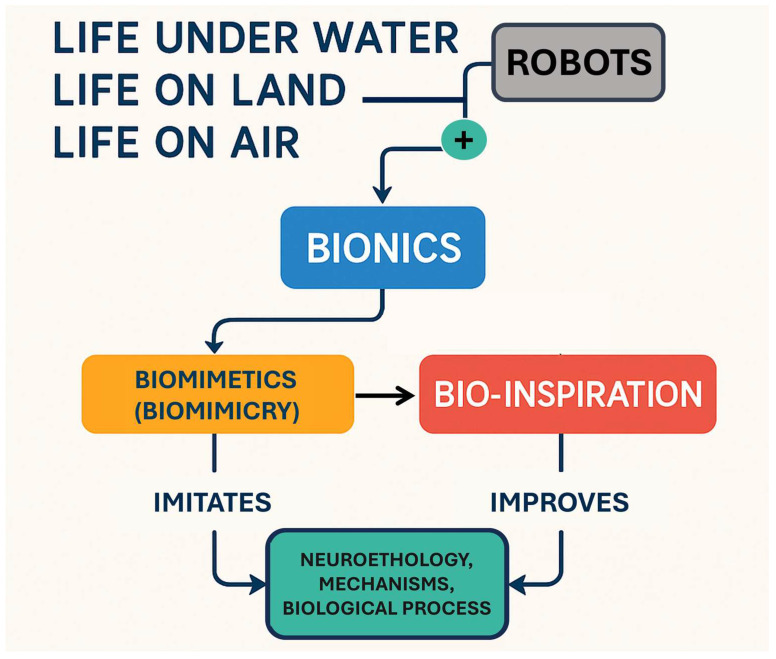
An overview of life’s diversity: exploring existence on land, air, and underwater, accompanied by technological and natural representations.

Over the last ten years, a profound synergy has emerged between biological systems and engineering innovation [24]. This interdisciplinary fusion is visualized through Figure 1, which captures the expanding scope of bio-inspired investigations. One of the most prominent developments has been the accelerating interest in bio-robotics and biologically inspired computational strategies [25]. This surge reflects a growing academic commitment to decoding and translating the refined efficiency of natural mechanisms, systems perfected through evolutionary processes over millennia, into adaptive technological frameworks. Meanwhile, regarding mechatronics design, since its conceptual inception in 1969 by Tetsuro Mori [26], and held as a trademark by a Japanese corporation until 1972 [27], it has undergone a profound evolution, maturing into a discipline defined by the seamless convergence of mechanical engineering, electronics, control systems, and information processing [28]. This integration has fostered the emergence of mechatronics systems, technological constructs wherein traditional mechanical functions are supplanted or augmented by electronically governed operations [29]. The result is a transformation of physical design: simpler mechanical architectures coupled with enhanced system intelligence and operational performance [30]. At the heart of mechatronics lies a philosophy of synergistic design, an approach that harmonizes structural mechanics, sensor-actuator integration, automatic data processing, and comprehensive system control. This synergy generates innovative functionalities and emergent behaviors unattainable through isolated disciplinary efforts [31]. Its impact is especially pronounced in the redesign of electromechanical systems, vehicles, precision instruments, and machines, many of which are now deeply intertwined with cloud computing and digital infrastructures [32]. Yet, with integration comes complexity. As mechatronics systems grow in sophistication, particularly in light of emerging paradigms such as cyber-physical systems and human-centered design [33], new challenges arise concerning system robustness, reliability, and cross-domain functionality. They necessitate the continual refinement of design methodologies, reaffirming the enduring relevance of the mechatronics model as a blueprint for multidisciplinary system integration [34]. To ensure coherence across engineering domains and prevent design conflicts, it is imperative to adopt rigorous methodologies that promote system-wide consistency throughout development [35]. Notable examples include techniques for verifying alignment between system requirements and design specifications [36], tools for integrating robotic components with embedded validation checking [37], and error detection [38]. It supports complex decision-making processes and facilitates traceability of structured knowledge, thereby empowering engineers to create mechatronics systems with precision, clarity, and coherence [39,40].

**Figure 2:**
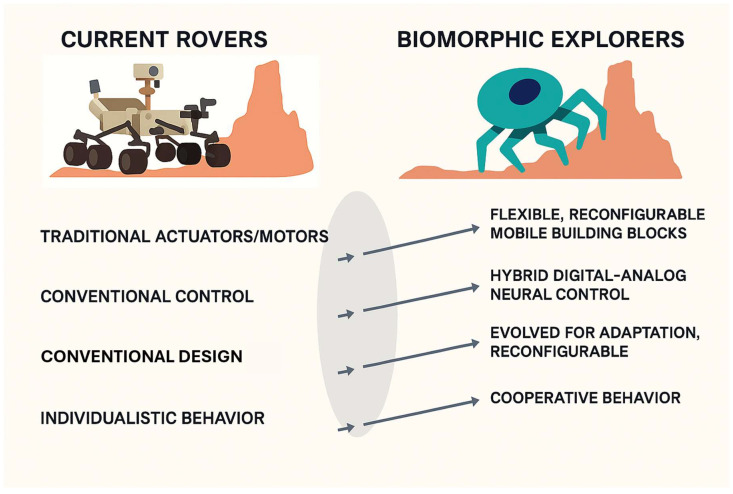
Common features of current vs. biomimetic space robots (planetary robots’ example).

Then, in the field of space technology design, biomimetics and bio-inspiration from nature’s ingenuity have emerged as a transformative paradigm, reshaping the conception of advanced space systems [41,42]. Recognizing the immense potential of biological-derived strategies, National Aeronautics and Space Administration (NASA) [43] and the European Space Agency (ESA) [44] conducted global research efforts aimed at translating biological principles into aerospace technologies. These explorations encompass not only the biomechanics of locomotion, such as walking, swimming, flying, grabbing-pushing, wriggling, undulating, burrowing [45] and rolling [46], but also extend to fundamental attributes including material composition, sensory integration, control techniques and behavioral adaptation [47]. Each of these dimensions holds profound relevance for extraterrestrial environments, where conventional engineering solutions are often inadequate [48]. The demands of space exploration impose severe constraints on system design. Exposure to radiation, atomic oxygen [49], extreme temperature fluctuations, vacuum conditions, and microgravity, all compounded by communication latency and the necessity for long-duration autonomy, renders traditional mechanical and electronic architectures vulnerable [50]. In contrast, biological organisms have evolved to flourish under similarly hostile and unpredictable conditions. Their inherent traits of adaptive capacity offer compelling models for the conception of space systems capable of enduring and excelling beyond Earth’s protective atmosphere [51], as shown in Figure 2. Autonomy remains a foundational requirement for space robotics and spacecraft systems operating far beyond immediate human oversight [52]. Bio-inspired control methodologies, including neural networks and reflex-based architectures, provide systems with the flexibility to adapt in real time to uncertainty and dynamic conditions [53]. Likewise, biologically inspired structures, such as compliant mechanisms (biogeometry [54]) and self-healing materials, endow spacecraft with the capacity to absorb impacts and recover from structural degradation during extended missions [55]. Such principles support a new generation of adaptive, robust systems designed for exploration in unstructured, off-world terrains [56,57]. To assess the viability of these technologies, performance evaluation must align with the exacting demands of space missions: energy efficiency, autonomous decision-making, multifunctional manipulation, and scientific instrumentation [58].

The central research question of this study is: How can biomimetic design methodologies be formally integrated with international engineering standards (VDI, ISO) and NASA TRLs to create a unified, verifiable pathway toward space-qualified robotic systems? The underlying hypothesis is that the proposed BEAM-D standard ensures that biomimetic principles can be systematically validated, benchmarked, and certified, transforming biological abstraction into flight-ready engineering solutions.

Therefore, this manuscript presents two foundational contributions to formally establish the discipline of Aerospace Bionic Robotics: (1) Strategic relevance of biomimicry in space contexts by showcasing how nature-derived principles, such as autonomy, adaptability, and multifunctionality, can address the extreme constraints of space systems. These principles, rooted in biological efficiency and refined by natural selection, offer a roadmap for creating designs that are not only structurally and functionally optimized but also inherently sustainable under the stresses of extraterrestrial environments. (2) Pioneering introduction of a comprehensive novel methodology for the design and integration of biomimetic systems within space technologies. By merging insights from biology and mechatronics, this framework supports a design process that is both systematic and creative. It enables the translation of complex biological strategies into engineered solutions that are manufacturable, scalable, and aligned with real-world mission requirements.

Finally, in order to introduce the BEAM-D standard, which is constituted by the BEAM-DX framework determines a modular implementation strategy structured into four progressive phases: BEAM-D1, BEAM-D2, BEAM-D3, and BEAM-D4 (see Section 4 for detailed explanation), that collectively guide the transition from space mission conceptualization to full-scale operational deployment. The remainder of the manuscript is structured as follows: Section 2 outlines design methodologies, categorized along the disciplinary axes of biomimetics and mechatronics. Section 3, upon these foundations, a novel methodology (BEAM-D) for Aerospace Bionic Robotics design, grounded in biomimetic principles, is proposed. Then, it is discussed in Section 4, contrasting the current knowledge gaps (based on manufacturing, interdisciplinarity, and evolutionary biology) with innovative benefits. Finally, the research concludes in Section 5 with a summary of key insights and directions for future research.

## 2. Engineering Design Standards: Technical Analysis

Constituting the backbone of translational biomimetics, this section provides the rigorous methodological scaffolding required to transform biological abstraction into verifiable mechatronic architectures capable of withstanding extreme aerospace environments. In the absence of such standards, the transfer of biological strategies risks remaining at the level of metaphor rather than achieving system-level integration, certification, and space qualification. The dual frameworks established by the VDI guidelines (6220–6226) and ISO/TC 266 [15] standards have therefore become indispensable, not merely as descriptive taxonomies but as normative instruments that guarantee terminological precision, methodological reproducibility, and cross-domain interoperability. Their relevance escalates when these biomimetic prescriptions are interfaced with mechatronics-specific standards such as VDI 2206 and the safety-centric protocols of ISO/TC 299 [15] for robotics, thereby unifying mechanical, electronic, and cyber-physical domains into coherent design pathways. In this section, we critically analyze these engineering standards and create structured diagrams as convergent instruments that enable the systematic embedding of autonomy, adaptability, and multifunctionality (core biomimetic principles) into the design of space mechatronic systems.

### 2.1. Biomimetic Systems

**Figure 3:**
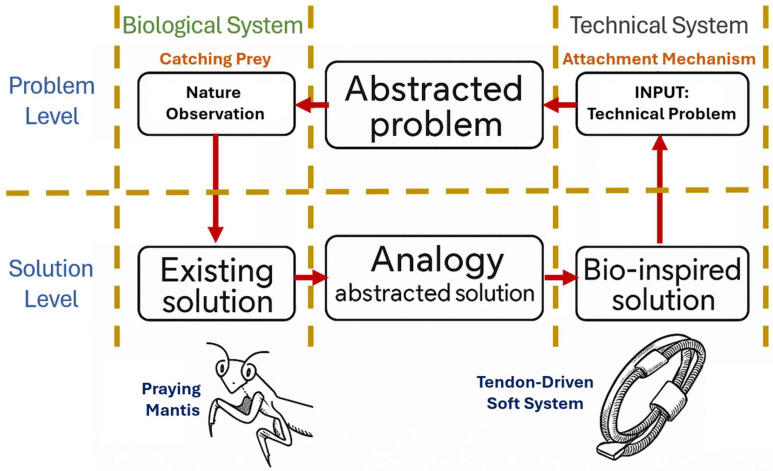
Analogical transfer in biomimetic design. It begins at the input stage, where a technical challenge is identified and abstracted into its essential formulation. This abstraction leads into the ideation stage, directing the inquiry toward the study of natural systems as a reservoir of morphological and biomechanical strategies. Through rigorous observation of biological organisms, fundamental principles and adaptive traits are extracted and reformulated. The process then advances to the creation of a technical solution, achieved through analogical abstraction that forges a bridge between biological insight and engineering application. The result is a bio-inspired solution, in which evolutionary knowledge is translated into engineering rigor and innovative design.

Several scholars have explored the dynamics of interdisciplinary collaboration between engineers and biologists [59]. The findings generally underscore the benefits of such partnerships. Notably, Helten et al. [60] reported that collaborative efforts led to more efficient and profound comprehension and assessment of biological systems by engineering teams. However, the research also highlights certain limitations. A recurring challenge lies in the disparity of communicative practices, differences in how information is structured, and the use of discipline-specific terminology, which often impede effective knowledge exchange [61]. The general knowledge of transfer thinking is shown in Figure 3, which shows that biomimetic design usually implies two sides: abstraction of the technical problem and abstraction of the biological solution [62].

**Figure 4:**
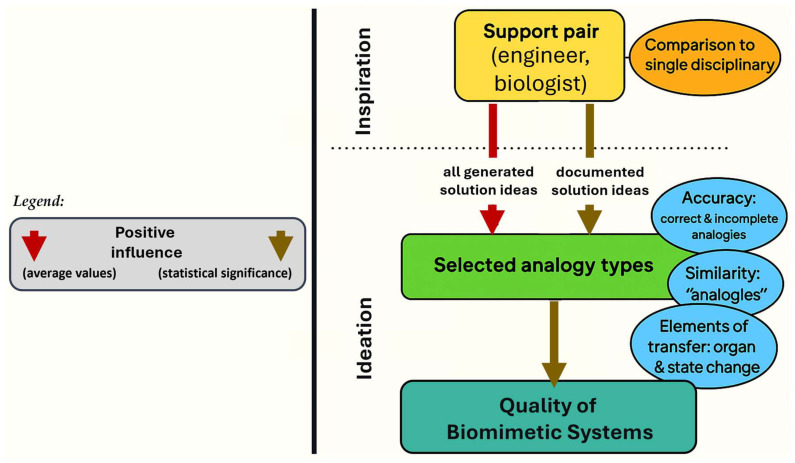
Inspiration and ideation process of biomimetic conceptualization. The process begins with a multidisciplinary collaboration between engineers and biologists, aimed at achieving the highest quality in biomimetic systems. This foundation rests upon accuracy, the formulation of analogies, and the structured use of transfer elements, which are represented both at the qualitative level (red rows) and the quantitative level (brown rows).

Biomimetic design seeks to translate biological analogies into groundbreaking technical innovations by harnessing nature’s proven strategies [63]. At its core, this approach prioritizes the generation of high-quality conceptual solutions, recognizing that impactful innovation stems from the strength of the initial idea. A fundamental objective in this process is to examine how various forms of bio-inspired analogies influence the caliber and creativity of the resulting technical concepts [64]. Given that nature offers a vast and diverse reservoir of inspiration, often spanning multiple species, this process naturally yields a wide array of potential solutions [65]. As illustrated in Figure 4, there are critical insights that emerge from this framework: (1) Robust methodological support facilitates the structured documentation of solution ideas aligned with specific types of analogies; (2) The intentional selection and application of appropriate analogical types significantly enhances the quality of conceptual outputs; (3) Systematic training in analogy transfer emerges as an effective strategy to strengthen biomimetic design capabilities; and (4) While collaboration between engineers and biologists proves beneficial, it necessitates targeted support mechanisms to bridge disciplinary gaps effectively. This multidimensional approach underlines the importance of structured reasoning, interdisciplinary synergy, and analogical precision in driving innovation through biomimetic methodologies [66].

In an effort to construct a comprehensive framework for biomimetics, the authors identified 24 fully accessible and operational tools (BIT-XX), detailed in Table A1 (Appendix A), which includes publication year, descriptions, nomenclature, and codes. As is common in engineering design, the proliferation of tools can create ambiguity, leaving practitioners uncertain about which tool is most appropriate for a given context [67]. A thorough examination of these tools reveals two principal origins (I) engineering, encompassing methods such as the 5-Whys [68] and the resolution of technical contradictions [69]; (II) biology, including frameworks like the 16 patterns of nature [70] and functional modeling [71]. Biomimetics fundamentally aims to dissolve disciplinary boundaries, not only by fostering dialogue between scientific domains but also by enabling the translation of theoretical insights from biology and engineering into practical design applications. Pursuing knowledge solely for academic ends can result in inefficiencies and misinterpretations in research and practice. Table A1 (Appendix A), therefore, leads to a critical inquiry: How might the existing theoretical infrastructure be refined to better facilitate the practical implementation of biomimetic principles? These tools form the structural foundation, the bones, of the biomimetic process.

The norm governing the field of biomimetics finds its foundational structure in the comprehensive set of guidelines issued by the Verein Deutscher Ingenieure (VDI) [72], specifically VDI 6220 through VDI 6226. These guidelines were meticulously developed by the Fachbereich Bionik (Biomimetics Group) under the auspices of the VDI Society for Technologies of Life Sciences. As the principal association representing German engineers, the VDI plays a pivotal role in providing professional frameworks, robust networks, and methodological standards that have significantly influenced the biomimetic discipline. These standards have proven especially influential in shaping terminology and establishing industrial applications, thereby laying the groundwork for international standardization. To that end, three ISO standards have been published under the umbrella of ISO/TC 266 [15], reinforcing global consensus on biomimetic practices.

Though the adoption of these standards remains voluntary, their value as practical guides in the implementation of biomimetics is undeniable [73]. They serve not only as methodological cornerstones but also as catalysts for scientific rigor and technological innovation within the discipline. This study seeks to explore how these standards are perceived within the engineering and scientific communities. Through a thorough analysis of scholarly publications and patent literature, this research finds the frequency and context in which these standards are cited, thereby providing insights into their practical impact. Furthermore, understanding user engagement with ISO biomimetic standards offers critical information for enhancing their dissemination and improving communication strategies across disciplines. Table 1 offers a consolidated view of the 14 principal guidelines and documents pertinent to this research, demonstrating that biomimetics is already well represented in the normative landscape. However, given the rapid evolution of this interdisciplinary field, it is anticipated that emerging topics will necessitate further standard development, thereby expanding the existing corpus of biomimetic regulatory literature.

**Figure 5:**
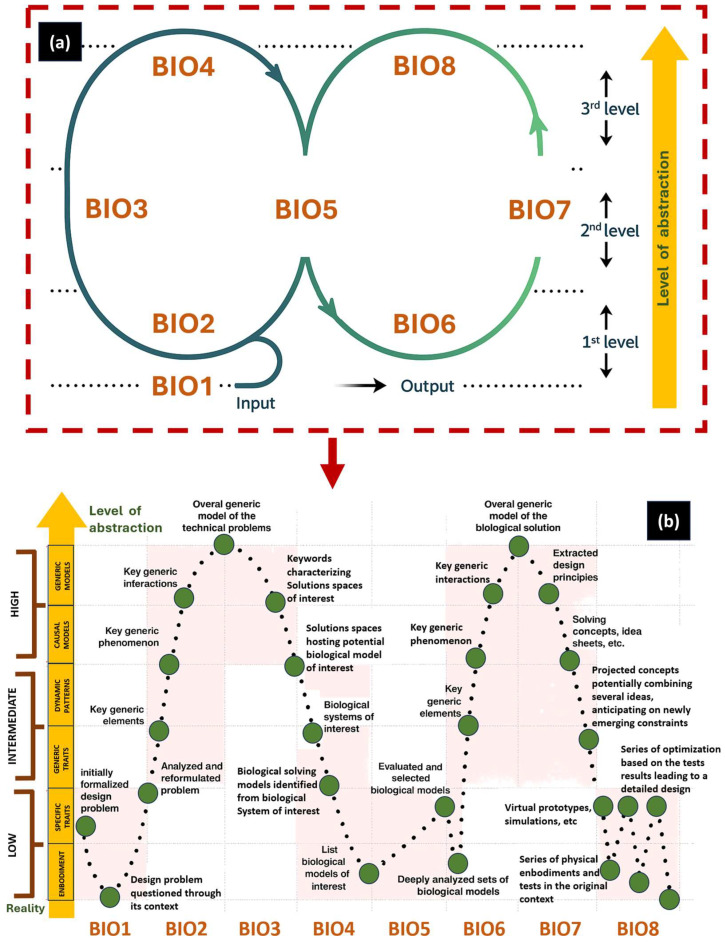
(**a**) Life cycle of biomimetic design (8 steps) starting in step BIO1 through BIO8. (**b**) The process is organized along the axis of abstraction, which delineates the progressive transformation from biological observation to engineering realization. Each component represents a distinct stage in the translation of natural principles into technical design.

According to Fayemi’s framework [88], it delineates the biomimetic design process into 8 fundamental steps, offering a structured methodology that serves as the foundation for the proposed refined biomimetic method in this manuscript. Within the practice of biomimicry, the process of abstraction emerges as particularly critical. In response to this challenge, Figure 5 proposes a redefinition and renaming of several procedural sub-stages. These revisions are grounded in both conceptual analysis and empirical insights. The objective is to enhance clarity and foster a deeper, more intuitive engagement with the biomimetic design process. To this end, the revised framework adopts a prescriptive tone, utilizing concise and action-oriented terminology designed to function as direct instructions. Such a linguistic shift is intended to support usability and accessibility, thereby empowering practitioners, regardless of disciplinary background, to navigate the complexities of nature-inspiration with greater confidence and precision.

As illustrated in Figure 5, the subsequent steps (BIOX) are outlined:

**Step BIO1:** Analyze the problem. The foundation of any biomimetic endeavor begins with a meticulous analysis of the context. While this step may appear conventional in design methodology, its pivotal role cannot be overstated. It provides the conceptual framework upon which all subsequent actions are scaffolded. For this reason, the process is formally initiated under the imperative directive of emphasizing its strategic and structural significance.

**Step BIO2:** Identify technical challenges and abstract their core dynamics. It demands the dissection of technical issues into their underlying patterns, causes, and effects. Often misunderstood when labeled as “abstraction of technical problems,” this step involves a systematic refinement and organization of data that defines the challenge. Practitioners transform problems into generalized conceptual patterns. These models reveal systemic interdependencies, preparing the research for cross-domain exploration.

**Step BIO3:** Transpose concepts into biology to reveal analogical solution spaces. It initiates a cognitive shift known as transposition, a process by which abstract, domain-independent concepts are projected from the technological realm into the biological domain. This analogical reasoning enables the identification of “solution spaces,” domains in nature where constraints mirror those of the technical problem [89]. These bio-spaces may be environmental, functional, or morphological. The involvement of biological experts becomes essential here, as organisms within these spaces are likely to embody adaptive strategies aligned with the design constraints.

**Step BIO4:** Conduct iterative search and identification of biological models. Initial searches generate new insights and terms that, in turn, refine the trajectory of further exploration. Each iteration enhances the specificity of solution spaces and raises the probability of discovering pertinent biological models. Although search and identification are occasionally treated as distinct sub-steps [90], their interdependence necessitates its integration into a single cognitive sequence, as practitioners constantly filter and refine search outputs to avoid informational overload.

**Step BIO5:** Evaluate and select the most viable biological model. Spanning multiple models is appraised against defined criteria. This step addresses a key difficulty observed among practitioners: the challenge of recognizing which biological insights are both functionally relevant and technically transferable. Selection results from comparative analysis, isolating those strategies that best fulfill the design objectives.

**Step BIO6:** Extract biological resolution elements. Once strategies are selected, attention turns to identifying the core resolution elements that define their function. These elements may include structural patterns, functional principles, or causal mechanisms. This distillation process is critical to ensure that only the essential, transferable aspects of the biological model are retained for application.

**Step BIO7:** Translate biological principles into technological design concepts. The abstracted biological principles are recontextualized within the technological domain. This is where analogical reasoning meets design creativity. The abstract insights serve as catalysts for ideation, enabling teams to generate innovative concepts with functional integrity. This step bridges the gap between biological inspiration and engineering application, effectively transmuting insight into action.

**Step BIO8:** Implement and validate through prototyping and testing. It grounds the conceptual output in practical execution. Design teams move forward with implementation, beginning with virtual simulations and advancing to physical prototypes. These iterations are tested rigorously to assess performance within the original problem context. Based on previous descriptive analyses, this step also unveils critical levers for process refinement and optimization.

**Figure 6:**
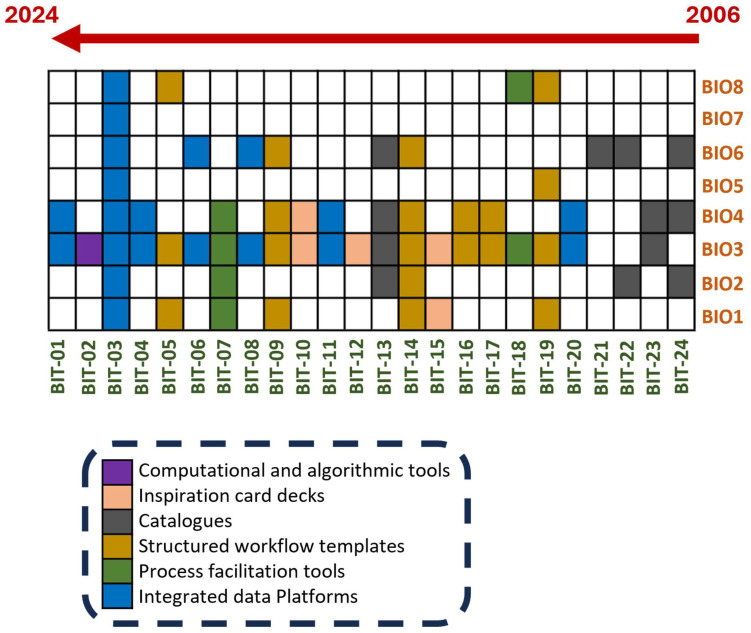
Relation between the 8-step biomimetic design methodology (BIOX) vs. 24 biomimetic tools (BIT-XX). The arrow above indicates the tool release timeline from 2006 to 2024.

Accordingly, Figure 6 depicts the proposed 8-step methodology (BIOX) that can be effectively integrated with the 24 biomimetic tools (BIT-XX) outlined in Table A1—Appendix A. The findings presented in this study offer an official snapshot of the current state of the field, acknowledging that the landscape of biomimetic design is likely to evolve in the coming years. By advancing the highlighted areas, the biomimetics community is well-positioned to cultivate new, impactful tools that not only enhance methodological rigor but also strengthen interdisciplinary collaboration and expand the practical application of nature-inspired innovations across diverse sectors.

### 2.2. Mechatronics Systems

**Figure 7:**
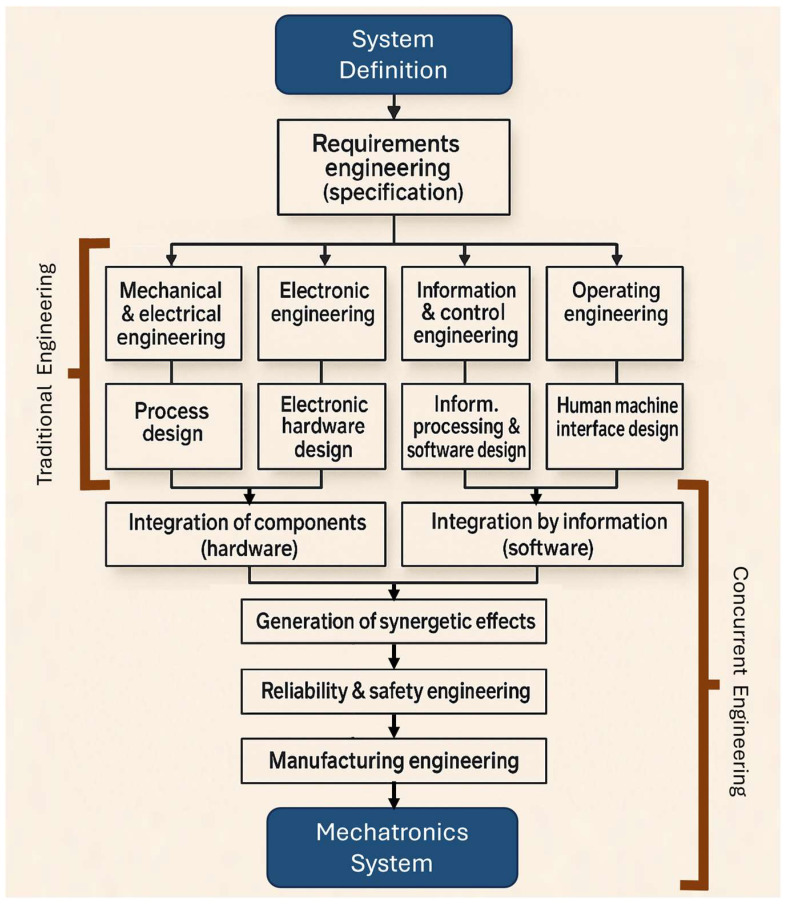
From domain-specific traditional engineering to an integrated mechatronics system.

This field represents a fusion of multiple engineering disciplines functioning cohesively. This multidisciplinary synergy typically involves the following: (I) mechanical engineering, which contributes physical components, mechanical devices, and precision mechanisms; (II) electrical and electronic engineering, providing microelectronic systems, power electronics, sensors, and actuator technologies; and (III) information technology, encompassing control systems, automation, artificial intelligence, cloud computing, and software development [91]. In mechatronics design, tasks are not confined to a single domain but are instead distributed across both mechanical and digital-electronic fronts. The bidirectional influence between these subsystems, where mechanical aspects shape electronic configurations and vice versa, necessitates concurrent engineering approaches (Figure 7). The objective is to foster a fully integrated system architecture, maximizing cross-domain synergy and enabling intelligent, responsive designs [92].

The core side of mechatronics lies in mechanical systems, which are foundational to a wide array of engineering applications and can be categorized based on structural features into general mechanical assemblies, micromechanical systems, and precision instrumentation. The creation of mechanical components involves managing the interaction of three core domains: energy, matter, and information. Depending on the nature of the engineering challenge at hand, one of these flows. Those typically tend to dominate the design process, with the others playing supporting roles [93]. At the heart of mechanical design lies the requirement to facilitate the controlled transfer of mechanical energy (forces and torques) to produce targeted motion profiles. Traditional design strategies involve considerations like material properties, mechanical strength calculations, manufacturability, and cost-effectiveness [94]. Historically, mechanical systems incorporated basic sensing and actuation elements to enable rudimentary control tasks. Over time, more advanced control mechanisms, including pneumatic, hydraulic, and analog electronic systems, were introduced. A pivotal shift occurred around 1975 with the introduction of microprocessors, which allowed for significantly more complex and intelligent control systems. Initially, these digital modules were retrofitted onto existing mechanical designs, but early implementations faced several limitations, including subpar durability in harsh environments (e.g., exposure to high temperatures, vibration, and contaminants), large spatial footprints, limited processing capacity, and extensive cabling [95]. However, advancements in electronics throughout the 1980s led to the development of highly compact, robust, and efficient hardware. With the integration of field bus architectures, engineers could reconceptualize the design paradigm: systems were no longer mechanical with electronic add-ons, but inherently mechatronics from inception. This led to distributed architectures, plug-and-play functionality, decentralized control, and modular, self-sufficient units (Figure 7), ushering in a new era of autonomous and intelligent mechatronics systems.

**Figure 8:**
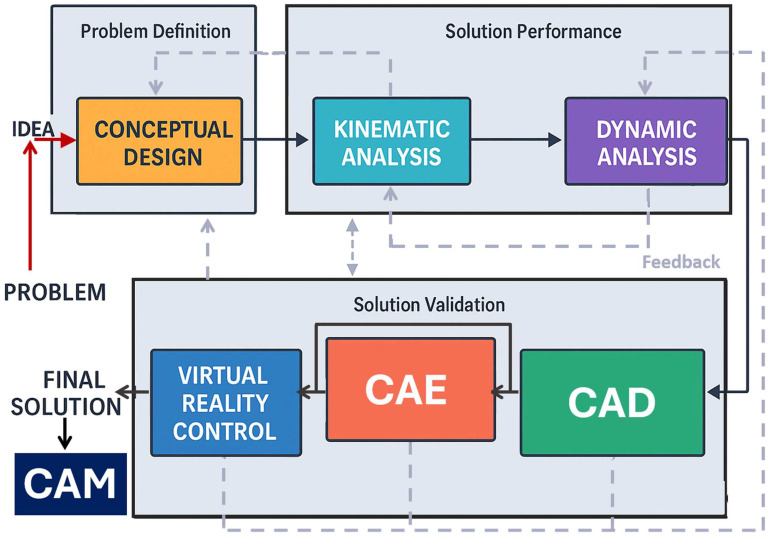
Foundations of mechatronics design methodology diagram. The process begins with a problem analysis (red arrow), leading to the formulation of a preliminary solution concept (input value). The forward pathway (black arrows) advances through three key stages: problem definition, solution performance, and solution validation, ultimately converging on a final solution that is subsequently prototyped using Computer-Aided Manufacturing (CAM). In parallel, the dotted grey arrows depict the feedback pathway, enabling the identification of potential refinements, detection of failures, and systematic optimization of the overall design process.

Technology development has introduced a method aimed at addressing the core challenges inherent in robotics [96]. Although fundamental domains such as kinematics, dynamics, and control have been thoroughly examined across diverse academic sources, these studies rarely align their focus with an integrated product development framework [97]. Frequently, the proposed approaches rely heavily on intricate analytical or computational models, which can be prohibitively complex for practical application by design engineers. Furthermore, essential stages in engineering development, such as conceptual modeling, Computer-Aided Design (CAD), Computer-Aided Manufacturing (CAM), and Computer-Aided Engineering (CAE), are being emphasized in most industrial and academic treatments of the subject [98]. Therefore, a foundational framework has emerged that systematically consolidates a variety of methodologies to address the robotic design process as an integrated, end-to-end workflow [99]. It encompasses all integrated steps, beginning with the initial problem statement and extending to the complete implementation of the robotic system. Significantly, it introduces forward-thinking strategies that leverage advanced digital tools to accelerate and enhance the solutions phases of the design process: definition, performance, and validation (Figure 8). Among its contributions are two key innovations: one involves the generation of tailored kinematic and dynamic models through the use of standard computational software (SCT-based mathematical modeling)-see Table 2, while the other offers a streamlined pathway for developing robotic prototypes by employing SCT within a non-mathematical, project management-oriented virtual environment.

Table 2 provides an overview of representative digital tools mapped to the principal domains that constitute a mechatronics system. In the realm of mathematical modeling, optimization and control platforms are frequently employed to analyze and simulate the behavior of dynamic systems. Complementarily, software environments for programming and implementing control logic are critical assets derived from the field of information technology. For electrical system development, simulation tools support the analysis of power electronics and instrumentation, including capabilities aligned with modern IoT integration.

Furthermore, artificial intelligence techniques are often embedded within this framework, enabling hybrid approaches that merge classical control strategies with heuristic-based decision-making algorithms [100]. Meanwhile, the physical and thermal dynamics of the system, including structural, fluidic, and environmental interactions, are addressed using CAD/CAM/CAE platforms, which underpin robust virtual prototyping and ensure performance validation before constructing the final hardware implementation.

**Figure 9:**
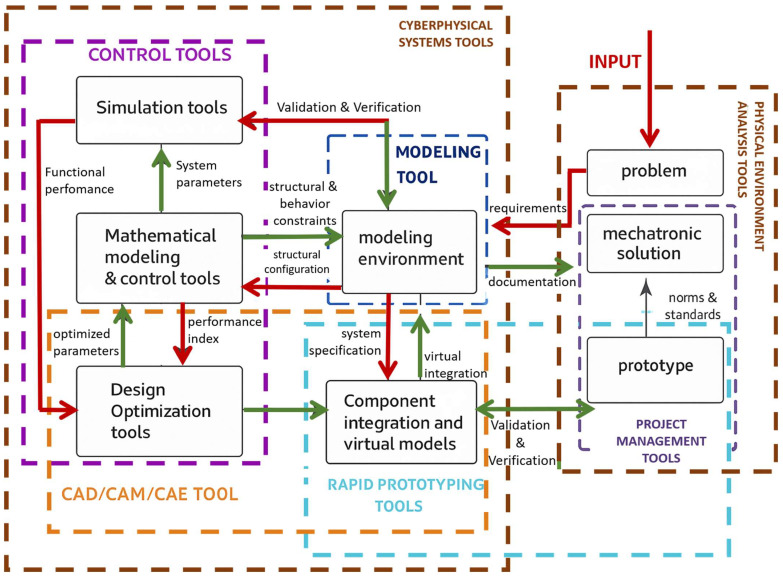
Global tools for the mechatronics design process. The workflow begins with the INPUT, where requirements and operational constraints are formalized and transferred (red arrows) to the problem definition and the modeling environment. The modeling stage integrates multi-domain representations, defining structural and behavioral constraints. Mathematical modeling and control tools generate governing equations and control laws, which are assessed in simulation tools for validation and verification, with performance indices fed back (red arrows). These results drive design optimization tools, refining design variables and producing optimized parameters (green arrows) that update the modeling cycle. The component integration and virtual models stage ensures subsystem compatibility through system specification exchange (green arrows). Rapid prototyping tools (blue arrows) translate validated models into physical prototypes for hardware-in-the-loop evaluation. Compliance with norms and standards ensures that the prototype advances toward a validated mechatronic solution. The grey arrow denotes maturity progression, from abstract problem formulation to a deployable product. Red arrows denote requirements and verification flow, green arrows parameter and documentation exchange, blue arrows virtual-to-physical transition, and grey arrows the degree of system maturity.

**Figure 10:**
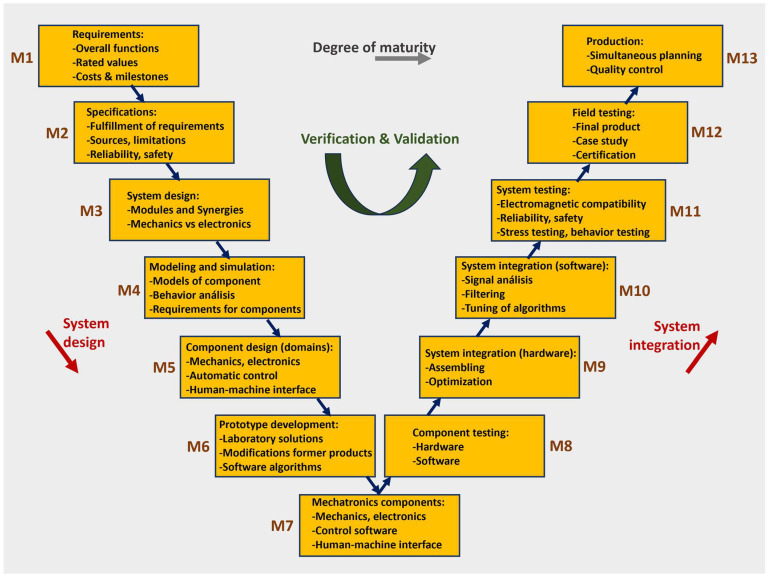
The V-model, as defined by the VDI 2206 guideline for mechatronic systems development, is represented in this diagram. The process comprises 13 sequential stages (black arrow), beginning at M1. These stages are structured into two principal blocks (red arrows): system design (M1–M7) and system integration (M8–M13). A continuous feedback pathway (green arrow) emphasizes verification and validation at every stage, ensuring systematic refinement and optimization throughout the process. Additionally, the grey arrow denotes the degree of maturity, progressing from left to right across the stages M1 to M13, with M13 culminating in a fully functional mechatronic product ready for end-user application.

Developing advanced mechatronics systems demands a structured design methodology supported by contemporary digital engineering tools. Unlike traditional mechanical or electrical design, the mechatronics approach is inherently more complex due to its interdisciplinary scope and iterative refinement process. Beyond the typical domain-specific engineering efforts, the development of such systems necessitates an integrated and concurrent engineering strategy. This approach reflects the deep interconnection across mechanical, electronic, and software disciplines, which defines the essence of modern mechatronics development. Historically, various engineering domains such as mechanical design, electronics, control systems, and human–machine interaction were treated as separate workflows, often handled by distinct departments with minimal overlap, frequently progressing in a linear, bottom-up sequence. However, the increasing need to harmonize both hardware and software elements mandates a more unified strategy. Now, these disciplines must operate in tandem to achieve a system-level optimum, following a concurrent, top-down design process. Such integration is most effective when executed by multidisciplinary teams. The recommended methodology for the development of this process is laid out in the VDI 2206 guideline [101], which proposes a flexible and modular design framework. As illustrated in Figure 9, it can be observed that there are 2 sides, the first where the input signal enters, is executed by “physical-environment analysis tools”, and the other is cyber-physical system tools. Both serve as the integrative nucleus of the entire development process, acting as the convergence point for all subprocesses defined within the V-model architecture (Figure 10). It functions as the central repository, continuously aggregating input data from a diverse array of engineering tools. These tools, each stemming from specialized domains within the design team, contribute iteratively to shaping the mechatronics system. All design iterations, technical decisions, and refinements are captured within this unified digital model, which governs the system’s progression from its initial conceptualization to its eventual physical prototyping.

As detailed in [102], the V-model serves as a key conceptual tool for organizing system design and integration activities across the mechanical, electrical, and software domains. This framework supports multiple design cycles and produces a series of intermediate deliverables, each reflecting a progressive level of maturity. These include: (I) Laboratory prototypes, which demonstrate preliminary functions, rough system architecture, and early-stage testing; (II) Functional prototypes, which incorporate refined components, energy management assessments, distributed system integration, and standard interfaces; (III) Pre-series models, which account for manufacturability, adherence to industry standards, modular consolidation, protective housing, and field validation. Although the V-model originated in the field of software engineering [103], it has been successfully adapted for complex mechatronics system development. Figure 10 illustrates an expanded version of this model, capturing three critical stages: system concept to lab model (M1–M7), integration through functional hardware-software definition (M8-M10), and validation leading to pre-production (M11–M13). Progressing through each step (MX) raises the overall product maturity. While not explicitly shown in the diagram, iterative loops are essential throughout the process. A fundamental premise of the V-model is the alignment between verification activities (right-hand side) and development processes (left-hand side), ensuring traceable documentation, robust testing, and technical consistency.

The extent to which mechatronics principles are applied varies depending on the nature of the product. In high-precision mechanical instruments, the fusion of mechatronics elements is often deeply embedded. For conventional mechanical subsystems, such as hydraulic brakes or adaptive dampers, traditional designs can be modified to incorporate actuators, sensors, and control electronics. In larger assemblies like industrial machines or vehicles, the core mechanical framework typically remains intact while being enhanced with mechatronics modules [104]. This hybrid approach is evident in modern robotics and computer numerical control (CNC) machinery, where intelligence and automation are layered onto robust mechanical platforms. The progression of hardware and software functionalities within such systems follows a defined steps (M1–M13) along the V-model structure (Table A2—Appendix A).

**Figure 11:**
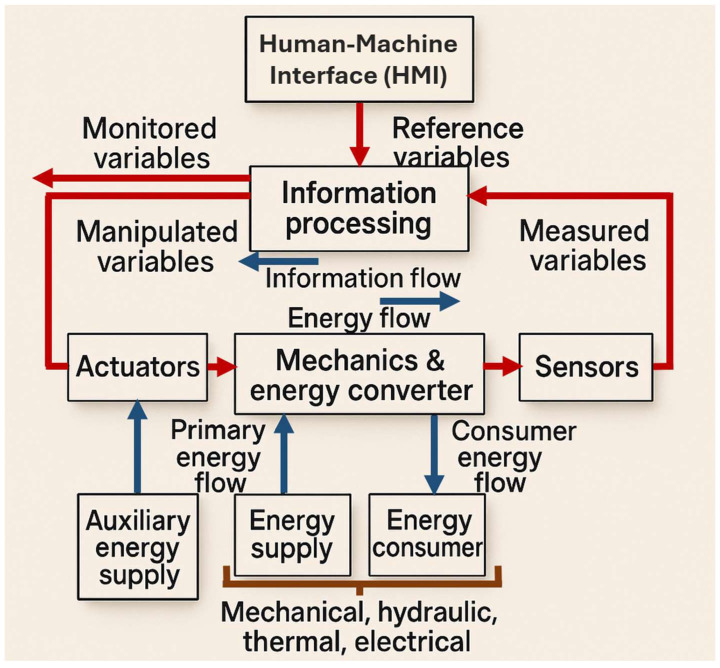
The diagram illustrates the functional architecture of a mechatronic system, beginning with the Human–Machine Interface (HMI), where reference variables are defined and transferred to the information processing unit, which generates the manipulated variables that drive the actuators and the mechanics and energy converter, ultimately interacting with the sensors, whose measured variables complete the closed-loop control cycle. The path of the red arrows denotes the flow of conceptual control variables, references, measurements, manipulated, and monitored signals, that govern the logic of regulation and supervision. In contrast, the blue arrows signify the flow of physical energy and structural information, extending from the primary and auxiliary energy supplies through the converters, consumers, and mechanical subsystems, where useful work is effectively produced. While the red pathway embodies the informational domain of control and decision-making, the blue pathway ensures the availability, transfer, and conversion of power. The integration of these two complementary flows guarantees that the operator’s intent is consistently translated into reliable and functional system performance.

A crucial focus within the specialized design step (“M5” of Table A2—Appendix A) is the development of the Human–Machine Interface (HMI)—Figure 11. This element is particularly vital in mechatronics applications for industrial and collaborative machines, including space robots, where the interaction between operator and machine must be intuitive and ergonomically optimized. In this domain, tools such as virtual reality and visual simulation platforms play a key role in refining interface designs and evaluating human-centric performance aspects, as emphasized in [105]. This context is highlighted by Daiker et al. [106], who present NASA’s application of Cognitive Task Analysis (CTA) to enhance the Graphical User Interface (GUI) of the Range Data Display System (RDDS), a pivotal component of the Launch Termination System (LTS). It is an integral real-time decision-making during launch operations, requiring rapid assessment of launch vehicle trajectories to ensure safety.

By dissecting the cognitive processes of Range Safety personnel, the Cognitive Task Analysis (CTA) identified user informational requirements, guiding the development of a more intuitive and efficient Graphical User Interface (GUI). This user-centered approach aligns with the VDI 2206 guideline for mechatronics systems design, which advocates for interdisciplinary collaboration and iterative development processes. V-model emphasizes the integration of mechanical, electronic, and software components through concurrent engineering and validation at each development stage. The incorporation of human factors engineering, as demonstrated in the CTA, exemplifies the guideline’s principle of considering user interaction as a core element of system design. Thus, the study not only advances the safety and reliability of launch operations but also reinforces the importance of human-centric design in complex mechatronics systems.

In addition, the ISO Technical Committee 299 (ISO/TC 299) [107] plays a crucial role in shaping the global development and deployment of robotics technologies. It establishes international standards that ensure safety and interoperability across a wide range of robotic systems. Key standards such as ISO 10218 [108,109] for industrial robot safety and ISO/TS 15066 [110] for collaborative robots are foundational to secure human–robot interaction in modern workplaces.

These guidelines provide systematic approaches to risk assessment, operational limits, and protective measures, thereby enabling safe cohabitation between robots and humans in shared environments. Beyond safety, ISO/TC 299 develops standardized terminology, classification methods, and testing protocols (Table 3). This fosters clear communication among manufacturers, regulators, and researchers, streamlining development and facilitating market adoption of robotic systems. Service robotics, mobile platforms, and autonomous systems all benefit from this unified framework.

## 3. Results: Design Standard for Aerospace Bionic Robots

**Figure A1:**
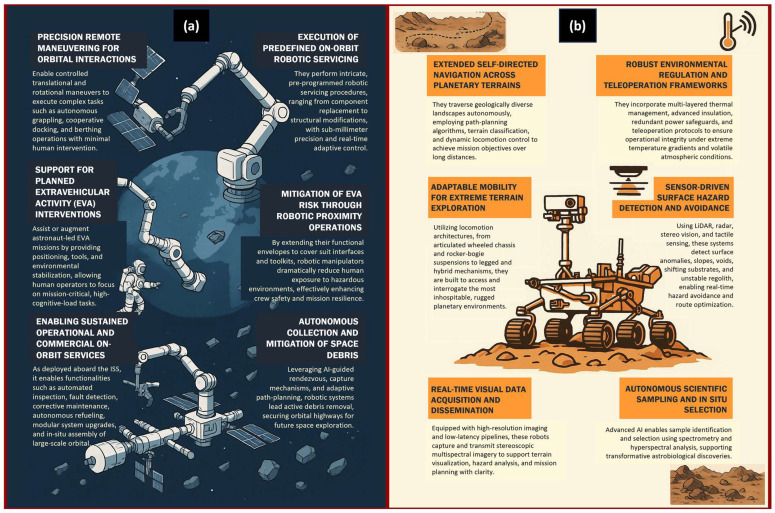
Task features of space robotics: (**a**) Orbital, (**b**) Planetary.

In the domain of space exploration, a diverse array of technologies has been developed to fulfill specialized functions, including various types of spacecraft (flyby, orbiter, atmospheric, lander, penetrator, rover, observatory, communications and navigation) [137]. Within this technological ecosystem, orbital robots (commonly referred to as orbiters), and planetary robots (widely known as rovers) stand out as key actors in advancing autonomous exploration missions (see Figure A1—Appendix A). These systems, when examined through the lens of next-generation design, are increasingly conceptualized using a synergistic integration of biomimetic principles and mechatronics frameworks as outlined in Section 2. This approach not only enhances their functional adaptability to extraterrestrial environments but also promotes efficiency in system behavior and autonomous decision-making. The purpose of Section 3 is to introduce a groundbreaking proposal BEAM-D: a standardized technical guideline for aerospace bionic robotics design focusing on mechatronics systems inspired by nature. This framework is rooted in biomimetic engineering methodologies, aiming to formalize the pathway by which intelligent robotic systems are conceived, prototyped, and deployed in space missions. By establishing a unified reference architecture, this standard is expected to streamline innovation while ensuring high performance, accuracy, and adaptability across diverse mission scenarios.

Since the early 1990s, the aerospace engineering community has recognized the critical need for standardized design protocols in space robotics, driven by the unpredictable and often hostile nature of extraterrestrial environments [138]. The necessity to create systems adaptable to diverse planetary terrains and vacuum conditions has long underscored the urgency of formalizing universal standards. As humanity ventures deeper into the cosmos, developing ever more sophisticated space systems under initiatives like the Space Exploration Initiative (SEI) and the former Space Station Freedom (SSF), the reliance on automation and robotic technologies is rapidly intensifying. These systems are no longer optional; they are essential. Long-duration missions with extended human presence demand autonomous mechanisms capable of performing complex maintenance, logistics, and operational tasks with minimal supervision. Automation and robotics (A&R) technologies are thus not only supporting tools but are becoming the backbone of extraterrestrial infrastructure [139]. Modern space robotics leverages hardware-software modularity, fault tolerance, advanced Robot Operating Systems (ROS) simulators, and reinforcement learning to enable autonomous operations in uncertain environments. However, standardization alone is insufficient. It marks only the initial step in the broader mission of ensuring functional and reliable robotic systems in space. True success lies in the deployment of mechatronics systems that merge mechanical intelligence with embedded computation, optimized for operation under extreme constraints [140]. Those, such as ultra-low mass, stringent energy limitations, and minimal human interaction, necessitate not only compact and efficient subsystems but also unprecedented levels of autonomy and artificial intelligence. In this context, space robots are engineered to perform tasks spanning from extremely hazardous operations to routine, repetitive functions, roles that are unsuitable or impossible for human astronauts [141]. From robotic manipulators assembling and maintaining orbital infrastructure [142] to autonomous explorers navigating the rugged terrains of the Moon and Mars, space robots are redefining the frontiers of extraterrestrial engineering. These systems must perform under extreme conditions, enduring severe temperature fluctuations, high radiation, and abrasive regolith. Operating in microgravity and without atmospheric protection, they require advanced mobility strategies, thermal regulation, and unprecedented levels of autonomy to thrive where humans cannot.

In the rapidly advancing domain of space exploration, the integration of automation and robotics standards is no longer optional but an indispensable cornerstone for the creation of autonomous, intelligent systems capable of executing interplanetary missions. Agencies such as NASA and ESA have acknowledged that conventional platforms like orbital and planetary spacecraft, while historically successful, are inherently constrained when operating in hazardous, irregular, or topographically inaccessible terrain. As scientific interest grows in these unstructured geographies [143], lava tubes, crater walls, and subsurface voids [144], the limitations of conventional mechatronics systems become apparent. To address these challenges, the field has pivoted towards biomimetic engineering, not simply mimicking morphology, but abstracting the evolutionary strategies that underpin nature’s most adaptable systems. This is the foundational principle behind the Biomimetic Engineering of Exploration Systems (BEES) initiative, introduced at Massachusetts Institute of Technology (MIT) in 2002, which posits that the hybridization of biological functionalities, such as proprioceptive navigation [145], transmedium exploration [146] (known as multi-domain locomotion), and multi-modal sensing [147], can enable biomimetic space robots to transcend the physical and computational barriers of conventional platforms.

This biomimetic transformation is tightly coupled with the evolution of robotics ontologies and interoperability standards developed by the IEEE Robotics and Automation Society (RAS) [148], such as IEEE 1872-2015 [149] and its extension IEEE 1872.2-2021 [150] formalize the Core Ontology for Robotics and Automation (CORA), which can be applied for mission-critical reasoning [151], situational awareness [152], and semantic knowledge representation [153] in deep space systems. In parallel with these advancements, IEEE 1873-2015 [154] and P2751 [155] provide standardized topological and metric mapping schemas in 2D and 3D, are key for precision navigation in GPS-denied environments [156] like Mars or the Moon, these standards are not abstract constructs; they serve as the digital scaffolding upon which intelligent space agents perform real-time Simultaneous Localization and Mapping (SLAM), object recognition, manipulation, and cooperative behavior in heterogeneous swarms [157].Reporting and critically analyzing all of the biomimetics and mechatronics standards mentioned in Section 2 and Section 3, we start introducing our proposed methodology of Biomimetic Engineering and Aerospace Mechatronics Design (BEAM-D). First of all, it is further strengthened by NASA’s PETAL (Prototype Engineering Tools for Agile Learning) framework and BIDARA (see Table A1—Appendix A, BIT-03), which instills a biologically inspired, iterative design methodology into the engineering lifecycle. PETAL leverages the cyclic structure of nature’s evolutionary refinement through its pillars: problem definition, exploration, testing, application, and learning.

Coupled with the Virtual Interchange for Nature-inspired Exploration (VINE), PETAL creates a distributed, cloud-based architecture for cross-disciplinary innovation, uniting clusters focused on big data, artificial intelligence (AI), machine learning (ML), sensors, and robotics [158], to coordinate a biomimetic intelligence loop, enabling rapid adaptation of robotic systems to multiple environments by incorporating real-time biological analogs and AI-informed decision layers. From a systems design perspective, this convergence gives rise to the new role of the Outer Space Product Designer (OSPD). In microgravity, the absence of terrestrial (the Earth) constraints liberates form and function, allowing for robotic architectures that morph, self-repair, and reconfigure. Additionally, in order to systematically translate biological models into engineered robotic systems, we consider the abstraction levels of VDI 6222 Part 1 (see Table 1), categorized into three principles: (I) Morphological: involves replicating biological form or structure, often through geometric or topological mimicry of anatomical features. (II) Functional: targets the transfer of mechanical or physical principles, such as locomotion mechanisms or energy conversion strategies found in nature. And (III) Behavioral: encompasses the replication of adaptive or responsive actions, including sensorimotor integration and environmental interaction.

**Figure 12:**
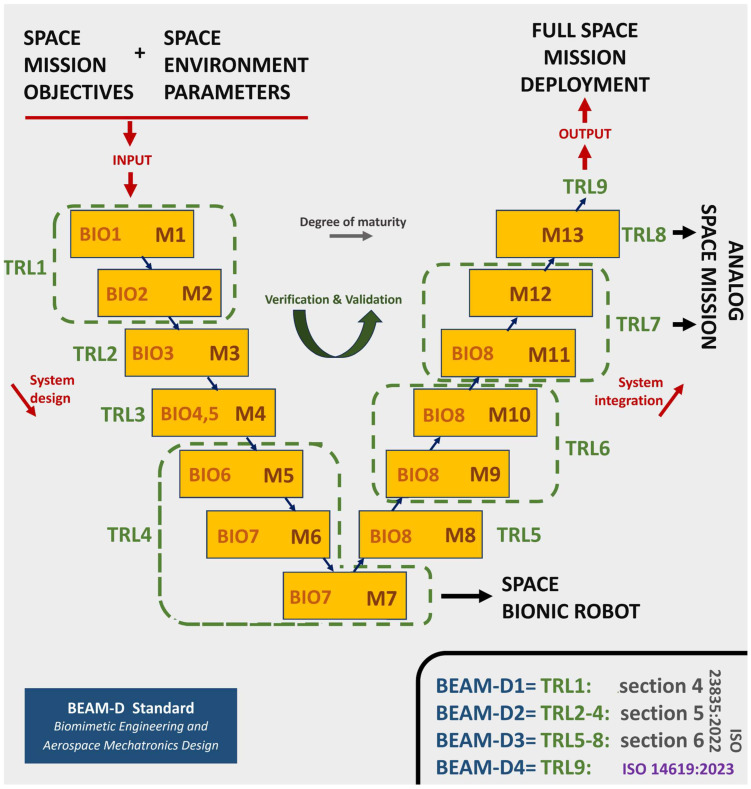
BEAM-D diagram (See Table A3—Appendix A) integrating the interactions across the axes: (a) TRL stages (tracking technological maturity); (b) VDI 2206/ISO standards (ensuring mechatronics and space technologies compliance and interoperability using ISO 23835:2022 [162] and ISO 14619:2023 [163])—see Table 1, Table 3 and Table A2—Appendix A; and (c) BIOX steps (guiding idea creation and refinement from a biomimetic background point of view)—see Figure 5 and Table A1—Appendix A. Note: TRL 1 (Concept Formation); TRL 2 (Technology Concept Validation); TRL 3 (Proof-of-Concept Demonstration); TRL 4 (Laboratory Validation in Controlled Environment; TRL 5 (Validation in Relevant Environment); TRL 6 (Demonstration in Relevant Environment); TRL 7 (System Prototype Demonstration in Space Analog Environment); TRL 8 (System Qualified for Flight); TRL9 (System Proven in Real Space Environment). The color coding of the arrows, with their meaning specified in the caption of Figure 10, should be considered.

In alignment with the Sustainable Development Goals (SDGs) set by the United Nations Office for Outer Space Affairs (UNOOSA) [159,160], the International Astronautical Federation (IAF) [161], and building upon the ISO 23835:2022 standard (which outlines rigorous protocols for the design, testing, and verification of robotic mechanisms in space systems) [162] and ISO 14619:2023 standard (Space systems experiments) [163], we incorporate a multi-layered systems engineering approach that synergizes Technology Readiness Level (TRL) measurements [164] with the structured logic of the VDI 2206 V-model for mechatronics development. This framework is further enhanced through integration with the iterative biomimetics design steps (BIOX)-Figure 5, enabling a cross-disciplinary convergence of biological principles and advanced robotics. The culmination of this hybridized model is embodied in the BEAM-D diagram, shown in Figure 12, which formalizes an innovative pathway for the co-evolutionary design and validation of bio-inspired robotic systems for extraterrestrial environments.

BEAM-D (Table A3—Appendix A) summarizes the development of biomimetic mechatronic systems progress through a structured sequence aligned with NASA’s Technology Readiness Levels (TLRs), beginning with conceptual problem definition, user requirements analysis, and abstraction of technical challenges into generalized solution strategies. Therefore (see Figure 12), our methodology enhances the integration of standards to be classified in 4 main phases called BEAM-DX: (a) BEAM-D1: Space mission overview, (b) BEAM-D2: Space bionic robot development, (c) BEAM-D3: Space bionic robot testing, (d) BEAM-D4: Space mission successfulness. Also, subsystem specifications are refined, components selected, and initial simulations validate functionality. Prototypes evolve from low- to high-fidelity, incorporating hardware-in-the-loop simulations and cyber-physical integration, enabling functional validation in controlled and simulated environments. Stress, thermal, and EMC analyses ensure effectiveness, while real-time calibration and human–machine interaction are optimized. Full system prototypes are tested under operational conditions, demonstrating feasibility, safety, and compliance. Final verification and validation are performed alongside manufacturing scale-up, ensuring readiness for deployment. The system achieves full operational capability through sustained engineering support, while the biomimetic methodology reinforces each step through iterative refinement, functional alignment, and continuous optimization grounded in biological insight.

In Table 4, Table 5 and Table 6, we have incorporated a structured framework that explicitly translates biological traits into measurable engineering parameters across all BEAM-D steps. Therefore, 3 project examples are described using information from space-tech resources in order to present the development and analysis of the optical geometry of lobster-eye, wood wasp ovipositor drill, and gecko-inspired adhesion as case studies. We quantify biological functions in terms of forces, stresses, geometries, and energy demands, ensuring reproducibility and traceability. Each BEAM-D step is mapped with specific objectives, modeling tools, component requirements, and standardized testing protocols, enabling a rigorous pathway from concept to analog validation. For example, gecko adhesion is parameterized in terms of adhesive stress, pad area, and detachment torque; lobster-eye optics are defined by pore size, aspect ratios, and angular resolution; while wood-wasp drilling is framed in torque, feed rate, and energy per depth. Acceptance thresholds, quantitative metrics, and TRL progression criteria are established, ensuring that biomimetic designs can be validated under laboratory, simulation, and analog environments. In this way, the BEAM-D standard provides a generalizable, quantitative, and standards-based process to systematically operationalize biological inspiration into engineering practice.

## 4. Discussion

This research examines the opportunity to engage the evolving opportunities that can provoke the transformative engagement of biomimetic design into higher-qualified mechatronic systems focused on enhancement, optimization, and novelty in robotic solutions, specifically in the aerospace sector. Biomimetic designers, through sequential systematic coding and adaptation of nature’s library of evolutionary adaptations, we have unlocked the BEAM-D standard that can generate the next generation of aerospace initiatives or facilitate entirely new engineering paradigms. Over the past few decades, biomimetic engineering has emerged as a powerful catalyst for disruptive innovation and sustainable development, especially in the creation of new manufacturing processes, intelligent systems, and models of interaction between humans and machines [198].

The path from biological inspiration to engineering conceptualization, moreover, is seldom a straightforward linear transposition, but rather often an overwhelming epistemological jump. There are often many biological strategies that are highly integrated and multi-functional and due to this, many do not have direct technological analogues to accommodate their learning, making the transition to engineered systems exceedingly difficult. As shown in Table A5 (Appendix A), a set of current challenges (State-of-the-Art Knowledge Gaps) constrain its full technical integration in the space robotics field. In recognition of this context, BEAM-D is seeking an increasingly, interdisciplinary research ecosystem involving a highly integrated self-organized teams of innovation [199]. Thus, as Wanieck [200] highlights, nature supplies context-specific solutions that require intelligent re-interpretation, novel material development, and contextual systems transfers. This limitation is further exacerbated by the scarcity of specialized design frameworks capable of identifying, evaluating, and translating biomimetic concepts into scalable and manufacturable engineering solutions. Such technical limitations become increasingly critical when engineering systems are intended for highly demanding operational conditions, observed in space environments, where structural and functional integrity often determine system survivability.

Biomimetics, therefore, represents far more than a repository of aesthetic inspiration; it constitutes a largely untapped vector for radical advancement. It requires an extremely deep systems-level understanding of biological processes, the advancement of next-generation research instrumentation, and manufacturing processes that can reproduce biological characteristics, and most importantly a paradigm shift to real interdisciplinary. Therefore, through the application of BEAM-D, the Aerospace Bionic Robotics development facilitates the convergence of biology, mechanics and material, electrical-electronics and computer systems, which we aspire to engineer aerospace systems that do not merely emulate biological principles but coevolve with humanity’s boldest aspirations, enabling the exploration and colonization of extraterrestrial settlements. Earth analog environments allow the testing of sophisticated robotic systems, developed for exploration of extra-terrestrial bodies (e.g., Moon, Mars, Titan, Europa). Land-based robots developed for lunar or Martian missions, were tested in either volcanic terrains or arid environments within the state of Hawaii or Utah, where they would be tested for autonomous navigation, mapping, and in-space resource utilization [201]. Notably, trials (see the details in Dataset A—Appendix A) can be conducted in Hawaiian volcanic terrains (Mauna Loa and Kīlauea), which provide basaltic substrates analogous to lunar and Martian regolith. During these tests, robotic subsystems were exposed to temperature fluctuations ranging from −11 °C to +40 °C, simulating the extreme diurnal thermal cycles of planetary surfaces. Additionally, at high-altitude volcanic sites (4205 m above sea level), prototypes were subjected to enhanced ultraviolet flux (11–12 or higher on the UV Index scale) and reduced atmospheric shielding, partially replicating the radiation environment encountered in orbit and on planetary surfaces [202]. Additionally, the Mars Desert Research Station (MDRS) in Utah, USA [203], serves as a robust terrestrial analog for validating space robotic systems due to several parameters that closely resemble Martian conditions. Its iron-rich, basaltic soils with fine particle sizes replicate the granularity, cohesion, and abrasiveness of Martian regolith, enabling realistic testing of drilling tools, anchoring systems, and rover mobility [204]. The site’s hyper-arid climate with very low humidity and wide diurnal temperature swings from −10 °C to +25 °C mirror the thermal variability of Mars (−153 °C to +20 °C) [205], providing a suitable environment for validating thermal insulation, electronic robustness, and energy storage subsystems. MDRS also presents fine airborne dust particulates, which, although generated under Earth’s 1 bar pressure, reproduce the abrasive and contaminant behavior of Martian dust that threatens optics, joints, and solar arrays [206]. Additionally, the layered sedimentary outcrops and basaltic ridges reproduce Martian geomorphology, offering challenging terrains for rover autonomy, navigation, and perception [207].

Aquatic habitats allow the testing of submersible robots with biomimetic propulsion, thermal drills, adaptive sensors for extreme pressure and low light conditions, all while simulating the icy oceans of Europa or surface lakes on Titan [208], and aerial drones or balloon platforms, in high-altitude or polar air environments, provide opportunities to mimic Titan’s thick atmosphere, and explore low-speed flight, long-duration flight, as well as atmospheric sampling [209]. The analog sites establish unique and critical physical and audience challenges for robotic missions and allow advance preparation of robotic autonomy, fault tolerance, and mission architectures before deployment to harsh off-world destinations.

Finally, to demonstrate the novelty of BEAM-D beyond assertion, a comparative benchmarking was performed against existing frameworks. Table 7 highlights the key dimensions across which BEAM-D advances the state of the art. Unlike ISO/IEC 15288 (general systems lifecycle), VDI 2206 (mechatronic V-model), or the Biomimicry 3.8 approach (biological-to-design translation), BEAM-D uniquely integrates TRL progression, iterative biomimetic steps (BIO1–BIO8), and BEAM-DX phases with Earth analog validation and space standards-aligned qualification.

Accordingly, bionic robots (biomorphic systems) are progressively reshaping the discipline of space engineering. Within this statement, the application of the BEAM-D standard is exemplified in 3 case studies (detailed and structured by each BEAM-D step in Table 5, Table 6 and Table 7: (I) The ovipositor of the wood wasp has inspired drilling tools suitable for subsurface planetary exploration. It can reduce required overhead force versus rotary systems and has been modeled/validated to need ~1.66 N·m drive torque at 2 m depth with peak cam torque about 2.5 N·m in regolith simulants, using a ~100 W motor; rotary drill torque requirements vary widely by design (≈0.3 N·m to tens of N·m) [165,210,211], since its initial conceptual translation into biomimetic drilling tools begins in the development phase but rapidly transitions into early-stage field testing) [166]. Also, (II) The unique optical geometry of the lobster-eye has guided the development of wide-angle, radiation-hardened imaging systems for astronomical observation. Modern micro-pore lobster-eye telescopes achieve fields of view up to 3600–4100 deg^2^ (~1.0–1.1 steradian-sr), exemplified by the Einstein Probe Wide-field X-ray Telescope (WXT). Operating primarily in the 0.5–4 keV soft X-ray band, these instruments deliver angular resolutions of 4–8 arcmin Full Width at Half Maximum-FWHM, enabling true focusing across very wide fields [212,213,214], as their implementation has advanced to laboratory-scale prototypes and field validation within optical payload assemblies [215]. (III) In microgravity environments, gecko-inspired adhesive mechanisms have enabled secure and controllable object manipulation, crucial for satellite maintenance and intra-vehicular operations. Engineered gecko adhesives can achieve pull-off strengths that vary by orders of magnitude depending on design and test conditions, with some reported values around 1.4 N·cm^−2^ and other engineered materials reporting much higher values in lab settings; and gecko-inspired grips have been successfully tested in microgravity (ISS and parabolic flight) [186,195,216,217], where the transfer of adhesion mechanisms into functional robotic end-effectors takes place [42,218]. Complementary studies, such as autonomous exploration robots with fish-inspired propulsion [219], and hierarchical control in swarm biomimetic platforms [220], further validate the need for integrated methodological frameworks aligned with TRLs and standards.

## 5. Conclusions

The exploration of biomimetic design for robotics and aerospace applications denotes both opportunities and challenges. Most systems are limited in terms of durability, self-maintenance, and self-cleaning capabilities necessary for most robotic applications. Overcoming these barriers could result in even more potential uses, particularly in astronomy, space and planetary exploration, on-orbit servicing and manufacturing, and maybe even debris reclamation. This paper has explored the elements of interest regarding biomimetic applications, especially within the context of space systems, detailing the appealing characteristics noted amongst biological systems employed when performing complex endeavours and their capacity for transferring those systemic characteristics into robotic applications. The method developed herein, BEAM-D, is a first step for developing biomimetic designs for space, whilst covering the TRL, VDI/ISO, and BIOX standards that would produce positive outcomes.

At the forefront of space robotics, biomimetics is ushering us into a new design paradigm where the design possibilities of human engineers will converge on the evolutionary “know-how” manifested by nature in order to overcome the challenges of exploration beyond Earth. As computational techniques expand and comparative intelligence continues to improve, it is now possible to both simulate and optimize complex natural systems with a degree of fidelity that has been previously unfathomable. This fidelity allows engineers to reproduce the functional sophistication of nature’s machines and deploy them via vehicles to outer space. Scientists can build planetary and orbiting robotic systems that adapt to space’s extreme environments, imitating adaptation and usability strategies developed by nature over billions of years. These advances push the limits of out-of-world mobility and autonomous exploration. However, it is not only the functional aspects of new technologies that need to be considered. Engineering progress itself acts as a wake-up call to emphasize responsibility. As we start to direct our exploratory efforts toward new worlds, we must avoid the same adaptation missteps that live-beings will endure. BEAM-D brings a philosophy of sustainability: efficient locomotion, low resource consumption, and entire lifecycle awareness. The space bionic robot of tomorrow is not just a marvel of engineering; it is a testament to our capacity to listen to nature, learn from it, and extend its harmony into the farthest reaches of space.

Furthermore, the variety of biological concepts offers a great opportunity for broadening adaptation for space technology. Certainly, there are well-documented challenges with biomimetic design, such as overcoming environmental constraints and reconciling the intended biological functions with engineered functions. Still, BEAM-D has the potential to be a rich source and an original pathway for innovative technical concepts and standards integration, improving or even revolutionizing traditional space robots. As this field develops, it will require further systematic study, continued development, and iteration to realize its full potential contribution to the creation of new aerospace technologies. In addition, BEAM-D is envisioned to extend beyond current applications, providing a framework for the creation of bionic robots inspired by both human and plant biomimicry foundations. This expansion highlights its versatility not only for extraterrestrial missions but also for addressing pressing challenges on Earth. By aligning with the Sustainable Development Goals (SDGs) promoted by UNOOSA [221], our methodology can guide the invention of bio-inspired robotic systems that contribute to sustainable industry, resilient infrastructure, and environmental stewardship. In this sense, BEAM-D stands as a bridge between advanced space exploration and the responsible use of biomimetic robotics for societal benefit.

This research has been rigorously developed within the domain of Engineering Design, specifically aligning with the recognized expertise fields of Design Theory, Design Methodology, and Design Cognition as articulated by the Technical Committee of the American Society of Mechanical Engineers (ASME) Design Engineering Division. The BEAM-D framework advances Design Theory by establishing formalized principles that unify biomimetic abstraction with standards-driven engineering protocols (VDI, ISO, and NASA TRLs), thereby extending theoretical constructs into new aerospace contexts. It contributes to Design Methodology through the systematic formulation of the BIOX cycle and BEAM-DX phases, which operationalize a reproducible process for translating biological strategies into validated mechatronic architectures, explicitly addressing traceability, standardization, and cross-domain interoperability. Finally, the work engages Design Cognition by integrating cognitive task analysis, analogy-based reasoning, and biological-to-engineering translation heuristics (Table A1, Table A2, Table A3, Table A4 and Table A5), thus demonstrating how designers can effectively bridge biological and engineering knowledge domains in high-stakes Aerospace Bionic Robotics applications. Collectively, these contributions situate the manuscript at the core of the ASME-recognized research trajectory in Engineering Design while introducing a first-of-its-kind standards-aligned biomimetic pathway for space systems.

## Figures and Tables

**Table 1:** List of VDI and ISO standards related to biomimetics.

Guideline	Code	Publication Year	Nomenclature	Reference
VDI	6620 Part 2	2023	Biomimetics—Biomimetic design methodology—Products and processes	[74]
VDI	6220 Part 1	2021	Biomimetics—fundamentals, conception and strategy	[75]
VDI	6224 Part 3	2017	Biomimetics—integrated product development process for biomimetic optimization	[76]
ISO	18457	2016	Biomimetics—biomimetic materials, structures and components	[77]
ISO	18458	2015	Biomimetics—terminology, concepts and methodology	[78]
ISO	18459	2015	Biomimetics—biomimetic structural optimization	[79]
VDI	6226 Part 1	2015	Biomimetics—architecture, civil engineering, industrial design—basic principles	[80]
VDI	6222 Part 1	2013	Biomimetics—biomimetic robots	[81]
VDI	6221 Part 1	2013	Biomimetics—biomimetic surfaces	[82]
VDI	6223 Part 1	2013	Biomimetics—biomimetic materials, structures and components	[83]
VDI	6225 Part 1	2012	Biomimetics—biomimetic information processing	[84]
VDI	6224 Part 2 withdrawn	2012	Biomimetic optimization—application of biological growth laws for the structure-mechanical optimization of technical components	[85]
VDI	6224 Part 1	2012	Biomimetic optimization—application of evolutionary algorithms	[86]
VDI	6220 Withdrawn without replacement	2011	Biomimetics—Conception and strategy—Differences between bionic and conventional methods/products	[87]

**Table 2:** Tools for mechatronics systems analysis.

Mechanics and Mechanisms	Electrical and Electronics	Control Techniques
SolidWorks	Proteus	Moveit.ai
Autodesk Inventor/Fusion 360	Autodesk Eagle	ROS/Gazebo
CATIA	PSIM	MATLAB-Simulink
Pro-E/CREO	NI Multisim	Labview
Autocad Mechanical	Modelsim	RoboDK
Siemens NX	Altium	Animatlab
Solid Edge	LTSpice	Webots
Ansys—LIGGGHTS/CFDEM	Ansys	Coppelia
COMSOL Multiphysics	Cadstar	OpenModelica
Working Model CAE	DesignSpark	SciLab—Xcos

**Table 3:** List of ISO standards related to robotic systems.

Guideline Code	Nomenclature	Reference
ISO/PAS 5672:2023	Robotics—Collaborative applications—Test methods for measuring forces and pressures in human–robot contacts	[111]
ISO 8373:2021	Robotics—Vocabulary	[112]
ISO/TR 13309:1995 ISO 9283:1998 ISO 9946:1999 ISO 14539:2000 ISO 9409-2:2002 ISO 9409-1:2004	Manipulating industrial robots	[113,114,115,116,117,118]
ISO 9787:2013	Robots and robotic devices—Coordinate systems and motion nomenclatures	[119]
ISO 10218-1:2025 ISO 10218-2:2025	Robotics—Safety requirements—Industrial robots	[108,109]
ISO 11593:2022	Robots for industrial environments—Automatic end effector exchange systems—Vocabulary	[120]
ISO/DIS 13482	Robotics—Safety requirements—Service robots	[121]
ISO/TS 15066:2016	Robots and robotic devices—Collaborative robots	[110]
	Robotics—Performance criteria and related test methods for service robots:	[122,123,124,125,126,127,128,129]
ISO 18646-1:2016	Part 1: Locomotion for wheeled robots	
ISO 18646-2:2024	Part 2: Navigation	
ISO 18646-3:2021	Part 3: Manipulation	
ISO 18646-4:2021	Part 4: Lower-back support robots	
ISO/DIS 18646-5	Part 5: Locomotion for legged robots	
ISO/DIS 18646-6	Part 6: Lower-limb wearable robots	
ISO/CD 18646-7	Part 7: Travelling around humans	
ISO/CD 18646-8	Part 8: Electric vehicle charging robots	
ISO 19649:2017	Mobile robots—Vocabulary	[130]
ISO/CD 21423	Robotics—Autonomous mobile robots for industrial environments—Communications and interoperability	[131]
ISO 22166-1:2021 ISO 22166-201:2024 ISO 22166-202:2025 ISO/AWI 22166-203	Robotics—Modularity for service robots	[132,133,134,135]
ISO/WD TS 25213	Robotics—Test methods for measuring the energy consumption of robots—6-Axis articulated industrial robots	[136]

**Table 4:** BEAM-D Case Study A.

BEAM-D Steps	PROJECT: Wood Wasp Ovipositor Drill
BEAM-D1	Mission Objective: Define required drilling depth, substrate(s) (regolith simulant, consolidated regolith/bedrock), sample volume, required sample preservation constraints, and energy/time budgets. Use the DROD literature guidance as a baseline, written by Alkalla et al. [165]: for small exploration drills targeting depths up to 2 m in consolidated simulants, design torque τ_required ≈ 1.5–2.0 N·m and continuous power budget P_drive ≈ 50–150 W depending on feed rate. For example, the referenced DROD study computed τ ≈ 1.66 N·m for 2 m depth and recommended a 100 W motor selection as practical. Feed rate v_feed design window: 0.1–5 mm·s^−1^ (slower for high cohesion substrates, faster for unconsolidated regolith). Energy per depth E_d (J·m^−1^) = P_drive/v_feed (J/m). Example ranges (P 50–150 W; v_feed 0.1→5 mm·s^−1^) produce E_d between ~10^4^ → 1.5 × 10^6^ J/m. Choose mission energy budget accordingly and size batteries/RTG/heaters with margin [166].
BEAM-D2	Use multi-physics co-simulation: structural FEA (bit and shaft dynamics) + transient thermal for bit heating + DEM for granular interactions, LAMMPS improved for general granular and granular heat transfer simulations (LIGGGHTS) [167] for coupling between the reciprocatory bit and granular simulant, where fracturing of consolidated substrate matters, couple FEA fracture models with quasi-static penetration simulations. For penetration into particulate regolith, calibrate DEM models with JSC-1A or MMS simulant physical tests. Motor selection: for τ_required ≈ 1.66 N·m and desired continuous operation, select a brushless motor with continuous torque rating ≥ τ_required × 1.5 margin (e.g., 2.5–3.0 N·m continuous). The DROD prototype used Maxon EC-i40 (in selection context) with gearing to reach the required torque. Provide output shaft gearing chosen for efficient speed/torque trade (gear ratio 10–30, depending on motor nominal torque) [168]. Bit design: segmented reciprocatory elements with hard coatings; design bit geometry for chip formation rather than pure abrasion; provide recommended material (tungsten carbide tip with Ni-coated shaft) [169] and design for bit replacement intervals (report MTTF in cycles from bench tests) [170].
BEAM-D3	Use JSC-1A (lunar) [171] or MGS/MMS 1–2 [172] (martian analog) simulants: for mobility and penetration tests; measure torque vs. depth, power vs. depth, energy per cm (J/cm), penetration rate (mm/s), and temperature at bit shank. JSC-1A is recommended by NASA for geotechnical tests [173]. Define energy per unit depth E_d (J/cm): measured under controlled feed rates. Example numeric results from prototypes: with P ≈ 100 W and v_feed = 1 mm·s^−1^ → E_d = 100,000 J/m = 1000 J/cm (→2 m requires ≈ 200 kJ). For slower feed rates (0.1 mm·s^−1^) energy per meter scales to 1,000,000 J/m (1 × 10^6^ J/m) [174].
BEAM-D4	Successful collection of samples to target depth with >80% mass/success retrieval across N = 10 replicate holes in JSC-1A; energy per depth within ±25% of model predictions; MTTF for bit and gearbox consistent with mission schedule. Passing analog trials with environmental exposure (dust, thermal cycling) supports TRL 6→7 progression [175].

**Table 5:** BEAM-D Case Study B.

BEAM-D Steps	PROJECT: Optical Geometry of Lobster-Eye
BEAM-D1	Mission Objective: Translate the biological lobster-eye concept into mission-level imaging metrics: required field of view (FOV), angular resolution (FWHM), energy bandpass, effective area, and detector spatial resolution. Adopt target imaging specs (example for a wide-field X-ray monitor): FOV ≈ 1.0 sr (~3600 deg^2^), angular resolution target FWHM ≤ 5 arcmin (choose 4–8 arcmin depending on trade). Energy bandpass typically 0.5–4 keV for soft X-ray monitors [176]. MPO geometric parameters to meet the imaging spec: select pore size d_pore = 10–20 µm and aspect ratio L/d ≈ 40–60 to produce a focused central spot and cross-arm signatures characteristic of lobster-eye optics (manufacturing and coating will affect reflectivity and effective area). Use glass micro-capillary or microchannel plate manufacturing with reflective coatings (Ir, Au, Ni) matched to the bandpass [177].
BEAM-D2	Optical/ray-tracing: use Geant4 (X-ray physics) or dedicated X-ray optics ray-tracers to model grazing incidence reflections, shadowing and effective area vs. energy. Combine Geant4 runs with structural-thermal-optical (STOP) analysis using ANSYS Mechanical (2022 R2) (Table 2) to set mechanical stiffness and thermal budgets required to preserve alignment to within optical error budgets [178]. MPO sector dimensions: choose sector plate thickness 1–3 mm (depending on stacking), open area ratio ≥70% preferred (maximize throughput), pore sizes selectable from vendors (6, 10, 20, 100 µm available), inner wall roughness < 1–2 nm RMS for high reflectivity [179]. Coating: Ir/Au/Ni coating thickness tuned to bandpass; coating process control must maintain roughness and avoid clogging pores [180]. Mechanical tolerances: manufacturing metrology should target per-sector alignment errors << 1 arcmin when possible (published manufacturing efforts show unslumped sectors approaching 1–2 arcmin alignment; current practice reports system FWHM ≈ 3–5 arcmin for flight MPOs). Use structural thermal optical performance (STOP) analysis to convert thermal/structural displacements [181] into angular resolution degradation—enforce that thermally induced relative displacements ≤ X µm [182].
BEAM-D3	Optical characterization: X-ray beamline testing (monochromatic energies across band) to measure PSF, FWHM, effective area vs. energy, and stray light (visible/UV transmittance must be <5 × 10^−4^ for MPOs with optical detectors). Record PSF maps and encircled energy fractions (EEF) at 50% and 80% [183]. STOP analysis steps: (1) thermal analysis for diurnal temperature swings expected in mission/analog; (2) structural FEA to compute deformations; (3) ray-trace those deformations to predict point spread function (PSF) degradation. Set acceptance: PSF FWHM degradation ≤ 20% of nominal; absolute FWHM ≤ mission spec (e.g., ≤5 arcmin). Use ANSYS + Zemax→ custom ray-trace coupling [184].
BEAM-D4	Full sector integration and in-vacuum X-ray beamline tests with measured FWHM ≤ mission threshold (e.g., ≤5 arcmin), measured Energy Efficiency Factor—EEF (50%), and effective area consistent with design to within ±15%. Passing these yields TRL 5–6 for the optical subsystem prior to environmental (vibration, thermal vacuum) testing [185].

**Table 6:** BEAM-D Case Study C.

BEAM-D Steps	PROJECT: Gecko-Inspired Adhesion
BEAM-D1	Mission Objective: Tasks requiring adhesion (e.g., free-flyer perching, docking/grappling of non-cooperative objects, anchoring for inspection/maintenance) and translate these tasks into force, moment, area, storage and environment requirements. Adhesive stress σ_adh target: adopt conservative nominal σ_adh = 1.4 N·cm^−2^ (14,000 N·m^−2^) for initial sizing; account for design-margin multiplier M_margin ≥ 1.25 when extrapolating to contaminated or worn surfaces. Use higher laboratory values (≈2.0 N·cm^−2^ = 20,000 N·m^−2^) for optimistic sizing when validated by ground tests [186]. Required adhesive area A_req (m^2^): compute A_req = F_required/(σ_adh × η_contact) where η_contact ∈ [0.6–0.95] is contact efficiency (accounts for surface roughness and partial contact). Example: to resist a 50 N inertial grab (example satellite capture load) with σ_adh = 1.4 N·cm^−2^ and η_contact = 0.8→A_req = 50 N/(14,000 N·m^−2^ × 0.8) = 0.00357 m^2^ = 35.7 cm^2^ (radius for a circular pad ≈ 3.37 cm) [187]. Detachment mechanics: if adhesive is peeled or deformed, design detachment actuator torque M_detach ≥ F_required × r_pad where r_pad is characteristic pad radius; for the example above r ≈ 0.0337 m → M_detach ≥ 1.79 N·m (before margin). Use actuator torque margin 1.5× to allow mechanical losses and misalignments [188]. Cycle life and endurance: plan N_cycles ≥5 × 10^4^ as a practical development target for adhesive microstructures in space (lab adhesives report tens of thousands of cycles under controlled tests; longevity must be validated per formulation) [186].
BEAM-D2	Use CAD → URDF for mechanical interfaces [189], and a physics co-simulation combining rigid multi-body dynamics (ROS/Gazebo or Ignition) (Table A4, Appendix A), contact model plugins with friction/adhesion models (custom Gazebo plugin or lookup table from bench tests). For adhesive contact mechanics, implement a directional shear-activated adhesion model (force vs. applied shear) parameterized by σ_adh, A_contact, and a detachment threshold; validate against peel/lap tests (ASTM D903/D1002) [190]. For autonomy and HIL, use ROS2 + Gazebo/ignition + ros_control.
BEAM-D3	Microstructure: microwedge length ≈ 80–120 µm, tip radius ≈ 0.5–1.5 µm (per microwedge PDMS geometries proven in ISS tests). Design wedge arrays to achieve A_d (contact area density) such that pad area × σ_adh ≥ F_required with margin [191]. Structural mounts: low-CTE interface materials (e.g., Ti-6Al-4V or carbon-fiber composite with matched mounting to adhesives) to limit thermally induced shear. Provide mechanical compliance (passive suspension) as recommended for Astrobee integration to ensure coplanar contact before loading [192]. Static pull-off (ASTM D1002 or similar lap shear/peel methods for comparative data): record normal pull-off stress (N/cm^2^) at 1 mm·s^−1^ separation, conditioned per ASTM humidity/temperature cycles [193]. Directional shear activation tests: measure shear-to-normal adhesion ratio; capture shear adhesive stress up to ≈ 80 kPa in microwedge tests (80 kPa ≈ 0.8 N·cm^−2^ shear stress reported in microwedge PDMS) [194].
BEAM-D4	Achieve on-orbit analogous perching/capture in lab/zero-g tests (e.g., gas-bearing rigs, parabolic flights, ISS demo) with successful perching/capture event rate ≥ 95% for test cases and survival of adhesives for ≥10^4^ cycles. The Astrobee tests achieved perching forces ≈3.15 N with pads launched in 2019 and stored on ISS for ≈1 year (demonstrating storage robustness) [195]. Environmental aging: gamma irradiation exposure and thermal cycling as in Day et al. [196]; validate adhesion retention after cumulative dose consistent with intended LEO/GEO/Martian exposure scenarios [197].

**Table 7:** Benchmarking related to BEAM-D.

Framework	Scope	TRL Integration	Biomimetic Focus	Validation Strategy
ISO/IEC 15288	Systems engineering lifecycle	No	None	Verification/validation steps
VDI 2206	Mechatronics V-model	Limited	Implicit	Model-based systems testing
Biomimicry 3.8	Design inspiration	No	Strong	Case examples only
BEAM-D	Biomimetic + mechatronic integration	Yes (TRL1–9)	Strong + structured	Case–controlled analog tests + TRL certification

## Data Availability

The original contributions presented in the study are included in the article. Further inquiries can be directed to the corresponding authors.

## Abbreviations

The following abbreviations are used in this manuscript:

A&R	Automation and Robotics
AI	Artificial Intelligence
BIDARA	Bio-Inspired Design and Research Assistant
BEES	Biomimetic Engineering of Exploration Systems
C-Dyn	Contact dynamics
CTA	Cognitive Task Analysis
CAD	Computer-Aided Design
CAE	Computer-Aided Engineering
CAM	Computer-Aided Manufacturing
CNC	Computer Numerical Control
CORA	Core Ontology for Robotics and Automation
DEM	Discrete Element Method
DVL	Doppler Velocity Log
EMC	Electromagnetic Compatibility
ESA	European Space Agency
GUI	Graphical User Interface
HIL	Hardware in the Loop
HMI	Human–Machine Interface
IAF	International Astronautical Federation
ISO	International Organization for Standardization
ISS	International Space Station
LiDAR	Light Detection and Ranging
MIT	Massachusetts Institute of Technology
MTTF	Mean Time To Failure
M/O	Mechanum/Omni
NASA	National Aeronautics and Space Administration
OSPD	Outer Space Product Designer
PETAL	Prototype Engineering Tools for Agile Learning
PDMS	Polydimethylsiloxane
RDDS	Range Data Display System
RAS	Robotics and Automation Society
ROS	Robot Operating Systems
SLAM	Simultaneous Localization and Mapping
SCT	Standard Computational Software
SEI	Space Exploration Initiative
SSF	Space Station Freedom
SDGs	Sustainable Development Goals
BEAM-D	Biomimetic Engineering and Aerospace Mechatronics Design
TRL	Technology Readiness Level
UNOOSA	United Nations Office for Outer Space Affairs
VDI	Verein Deutscher Ingenieure
VINE	Virtual Interchange for Nature-inspired Exploration

## Appendix A

The presence of Table A1, which systematically organizes the 24 biomimetic tools applied for design, is of foundational importance to the integrity and scientific depth of the manuscript because it functions as the epistemological core of the BEAM-D standard, where those tools have been valuable to define BIOX (see Figure 6). In biomimetics engineering—particularly when extended to aerospace mechatronics—progress is not only dependent on conceptual abstraction but on the explicit and reproducible documentation of the heuristics, analogies, and biological transfer mechanisms that underpin technical solutions. Table A1 provides precisely this function by consolidating the full spectrum of biomimetic tools employed, ranging from functional abstraction and top-down biological analogy mapping to advanced bio-inspired optimization and multiscale simulation techniques. This structured overview ensures that the methodology is not a black box but a transparent, standardized design pipeline where every engineering decision can be traced back to its biological or systemic origin.

From a strictly engineering design perspective, the tools compiled in Table A1 are of critical technical importance because they formalize the translation of biological principles into quantifiable engineering solutions. Techniques such as functional abstraction, analogy-based reasoning, multi-criteria optimization, and morphological analysis do not simply inspire concepts but provide rigorous frameworks for transforming biological strategies into validated design architectures, particularly under the extreme constraints of aerospace applications. In the context of space mechatronics, where safety, reliability, and performance validation are paramount, Table A1 demonstrates that biomimetic approaches can be integrated into engineering workflows with the same rigor as traditional systems engineering methods. Its inclusion transforms the manuscript into a methodological compendium and reference toolkit that future researchers and practitioners can directly adopt, replicate, and extend.

**Table A1:** Overview of the 24 biomimetic tools used for design.

Reference	Published Year	Description	Nomenclature	Assigned Code
[222]	2024	A web-based platform engineered for taxonomy-structured exploration of biodiversity datasets, enabling bio-inspired design through access to functional examples, species interrelations, ecological contexts, and integrated scientific resources. https://bioinspire-explore.mnhn.fr/ (accessed on 24 May 2025).	Bioinspire- explore	BIT-01
[223]	2024	A specialized search engine developed to retrieve biomimetic data using semantic web technologies and data-centric programming, increasing retrieval precision and contextual relevance for bioinspired research queries. https://github.com/emunatool/BARcode-BioInspired-Search (accessed on 24 May 2025).	BARcode	BIT-02
[224]	2023	An AI-powered assistant (ChatGPT by OpenAI), deployed by NASA, that supports biomimicry research by leveraging interactive dialogue to integrate biological strategies with sustainability frameworks from the Biomimicry Institute. https://github.com/nasa-petal/bidara (accessed on 24 May 2025).	BIDARA (Bio-Inspired Design and Research Assistant)	BIT-03
[225]	2023	An AI-augmented retrieval platform that extracts biologically pertinent analogs from a corpus exceeding four million peer-reviewed publications, now subsumed into Asteria for high-level biomimetic innovation workflows. As of January 2025, BiOMIg Search has been consolidated into Asteria, an advanced professional platform for biomimetic research and design. For further details, consult the Asteria system interface.	BioMIg search	BIT-04
[226]	2023	Provides a design framework for applying biomimicry principles through a flexible, nonlinear process, enabling designers to incorporate nature’s functional strategies into engineering or architectural innovation. https://biomimicry.net/the-buzz/resources/biomimicry-designlens/ (accessed on 24 May 2025).	Biomimicry DesignLens	BIT-05
[227]	2022	An online archive aggregating biomimicry literature, intellectual property, and peer-reviewed studies, offering researchers a curated knowledge base for developing nature-inspired solutions across technical domains. www.lib.mimic.us/ (accessed on 28 July 2025).	MIMICUS library	BIT-06
[228]	2021	This digital tool facilitates cross-disciplinary collaboration by standardizing data representation methods used by biologists and engineers for technical abstraction and modeling of nature-derived problem-solving strategies. https://linkage-lcpi.com/ (accessed on 28 July 2025).	LINKAGE	BIT-07
[229]	2021	A discovery platform providing biological model references and green design inspiration, featuring a comprehensive database of biomimetic cases and foundational knowledge for sustainable development. www.mimic.us/ (accessed on 28 July 2025).	MIMICUS data cloud	BIT-08
[230]	2020	An interdisciplinary interface for translating biological analogs into technical concepts, enhancing the novelty and feasibility of bio-inspired innovations across engineering design workflows. https://www.bioid.com/ (accessed on 28 July 2025).	BioId Support	BIT-09
[231]	2020	A technical repository of biological morphogenesis examples to support the creation of adaptive interfaces, facilitating integration of biomimicry in HCI, soft robotics, and responsive materials research. https://usabilitypanda.com/morphino (accessed on 28 July 2025).	Morphino	BIT-10
[232]	2019	A search utility enabling functional co-occurrence queries within the AskNature dataset, designed to assist engineers in identifying and applying relevant biological strategies to complex design challenges. http://biomole.asknature.org/ (accessed on 28 July 2025).	Biomole	BIT-11
[233]	2018	An educational tool comprising 24 biologically inspired challenge cards, designed to cultivate innovation through cooperative problem-solving and applied creative reasoning in biomimicry. https://biomimicards.com/ (accessed on 28 July 2025).	Biomimicards	BIT-12
[234]	2018	Employs a semantic translation engine that converts engineering requirements into biologically indexed terminology, streamlining access to functional analogs for concept generation in biomimetic design. www.uakron.edu/bric/E2BMO/index.html (accessed on 30 July 2025).	E2BMO	BIT-13
[235]	2017	Based on Concept-Knowledge (C-K) Theory, this methodological canvas provides a structured innovation process that supports bio-inspired ideation and transdisciplinary integration of biological knowledge. https://jacquelynnagel.com/bid-canvas/ (accessed on 30 July 2025).	BID Canvas	BIT-14
[236]	2016	Instructional cards are used to introduce biomimicry through structured knowledge transfer, allowing users to examine biological functions and their engineering applicability across multiple innovation domains. https://www.biomimicry.net/product/resource-cards/ (accessed on 30 July 2025).	Biomimicry 3.8 cards	BIT-15
[237]	2015	A digital compendium documenting biological systems and corresponding functions, enabling systematic analogical reasoning and supporting biomimetic ideation through structured classification frameworks.	BioCards	BIT-16
[238]	2015	An integrated modeling approach that combines biological and engineering paradigms using diagrammatic tools to enhance interdisciplinary communication and innovation in technical product development.	KoMBi	BIT-17
[239]	2015	Outlines 10 overarching biological principles to inform biomimetic design, ensuring system compatibility with ecological parameters and alignment with life-supporting natural patterns. https://toolbox.biomimicry.org/core-concepts/natures-unifying-patterns/ (accessed on 30 July 2025)	Nature’s unifying patterns	BIT-18
[240]	2014	The T-Chart framework aligns engineering problems with biological analogies by systematically correlating design parameters, enhancing analogy evaluation and solution applicability. The four-box framework organizes biologically inspired design problems by delineating function, environment, design constraints, and performance metrics to improve problem specification.	T-chart and Four-box method	BIT-19
[241]	2013	A centralized repository offering over 1700 nature-inspired strategies and cases, providing technical insight for developing biomimetic innovations across design, engineering, and sustainability fields. https://asknature.org/ (accessed on 30 July 2025).	AskNature	BIT-20
[242]	2011	Translates technical design language into biologically relevant keywords, enabling more effective search and discovery of functional analogs for biomimetic solution development.	Biologically meaningful keywords	BIT-21
[243]	2010	Converts biologically encoded mechanisms into engineering-relevant constructs, fostering reciprocal understanding and enabling biologically informed design implementation.	E2B-thesaurus	BIT-22
[244]	2008	Functional taxonomy tool that classifies biological strategies by purpose, simplifying access to biomimetic insights and enhancing AskNature’s utility for function-driven innovation. https://asknature.org/resource/biomimicry-taxonomy/ (accessed on 24 May 2025).	Biomimicry Taxonomy	BIT-23
[245]	2006	Synthesizes TRIZ inventive principles with biological strategies, providing a structured approach for generating biomimetic solutions via analogical reasoning rooted in natural systems.	BioTRIZ	BIT-24

As the gold standard for structuring product development in mechatronics, VDI 2206 provides not just a sequence of stages, but a conceptual framework that integrates mechanics, electronics, and control into a coherent development process. Table A2 distills this into clear, actionable steps, bridging abstract methodology with practical engineering execution. Its significance extends beyond procedural guidance: it offers a blueprint for reproducibility, reliability, and innovation, guaranteeing that the design flow accounts for cross-domain interactions, iterative validation, and integration challenges, which demand high importance in the context of space mechatronics.

**Table A2:** Analysis of steps included in VDI 2206 for mechatronics systems.

Steps	Key Features
M1. Requirements	User requirements are identified, including desired product functionality, performance benchmarks, and expected outcomes. A conceptual framework and solution strategy are developed, with initial cost estimations and a project timeline defined. Key milestones are planned. The output is a detailed requirements document, excluding specific technical implementations, serving as a foundation for subsequent steps in the mechatronics system development lifecycle.
M2. Specifications	The product concept is developed to meet user requirements, broken down into functional modules with defined specifications. Evaluation of tools, limitations, and resource availability is conducted. Hardware components are selected, and software environments, including compilers and development platforms, are specified. The result is a complete technical specification document, aligning multidisciplinary design efforts across mechanical, electrical, and software domains.
M3. System design	Subsystems: mechanical, electronic, hydraulic, pneumatic, and thermal, are detailed with power demands. Sensors and actuators are specified, including parameters and interface definitions. Intelligent component tasks are assigned. Hardware includes microprocessors, bus systems, and wiring. Control design addresses open- and closed-loop strategies. Models support calibration and diagnostics. CAD assesses reliability. The outcome is a fully documented, integrated control system design.
M4. Modeling and simulation	Mathematical models (kinematics and dynamics) for system components are developed using theoretical and empirical methods. Modeling tools and identification techniques are applied. Experimental setups and testing protocols are created (CAE). Model types include statics (forces, torques, geometries) and dynamics (position, velocity, acceleration, mass, stiffness, damping, friction, and input forces). The result is a set of validated models describing the physical processes and components, enabling accurate behavior prediction.
M5. Component design (domains):	Mechanical, sensor, actuator, and electronic designs are finalized. Hardware and software integration is performed. Functional prototypes are developed, emphasizing system functionality. Human–machine interfaces are designed for effective interaction. Initial testing is conducted using laboratory models. This step ensures that mechatronics components are operable and prepared for integration, supporting progressive system validation through user-centered, iterative development practices.
M6. Prototype development	This step involves creating cyber-physical models to test core concepts, evaluate interactions between mechanical, electrical, and software components, and identify design flaws early. It enables iterative refinement, fosters interdisciplinary collaboration, and reduces development risks, leveraging CAM tools to gain tangible insights before committing to full-scale production or system integration.
M7. Mechatronics components	All mechanical and electronic components are physically assembled. Sensors, actuators, and microcontrollers are integrated with structural elements. Electrical systems, cabling, switches, and communication buses are connected. This step ensures full hardware integration and operational readiness. The outcome is a functional mechatronics prototype, enabling system-level testing, iterative refinement, and seamless transition into performance evaluation and reliability assessment stages.
M8. Hardware and software testing	Hardware-in-the-loop simulations replicate the real-time behavior of the system. Software performance is evaluated under realistic operational conditions. Mechanical structures undergo stress and thermal analysis. This step ensures both digital and physical systems perform reliably, accounting for environmental challenges and failure modes. The prototype’s resilience is quantified, supporting safe, long-term deployment in dynamic or safety-critical mechatronics applications.
M9. Final system integration (hardware)	Final assembly incorporates mechanical and electrical adaptations. Components are aligned for functional and spatial compatibility. Manufacturing processes are optimized (CAM), improving cost and production efficiency. Component synergies are exploited to reduce redundancy and enhance integration. This ensures the transition from prototype to a manufacturable system is smooth, aligning design objectives with industrial standards and scalable production strategies.
M10. Final system integration (software functions)	Real sensor and actuator signals are used to calibrate system responses. Signal conditioning and filtering enhance data quality. Control parameters are fine-tuned based on test results. System performance is verified under steady and transient states. Human–machine interaction is validated for usability. This step ensures precision, reliability, and user safety before final product testing and certification.
M11. System testing	Test benches simulate operational stresses. Components are exposed to extreme conditions to verify endurance and durability. Safety and reliability metrics are assessed against predefined thresholds. Electromagnetic compatibility (EMC) testing ensures system integrity across noisy environments. These evaluations validate the mechatronics system’s robustness, preparing it for regulatory approval and real-world deployment in demanding application environments.
M12. Field testing	Final evaluations simulate normal and failure scenarios. System behavior is statistically analyzed for consistency and reliability. Certification processes are initiated based on regulatory standards. The results confirm product compliance, functional safety, and readiness for market entry. This step consolidates design objectives and assures stakeholders of the system’s performance in varied, real-world use cases.
M13. Production	Production infrastructure is planned and established. Manufacturing equipment is selected and configured for optimal throughput. Assembly lines are laid out to streamline component integration. Quality control systems are implemented to ensure repeatable product performance. This step translates design into scalable manufacturing, supporting consistent production of high-quality, reliable mechatronics systems ready for commercialization and mass deployment.

Table A3 is crucial because it formalizes the BEAM-D standard by aligning biomimetic design steps with NASA’s TRL framework and the BEAM-DX phases. It provides structured progression from problem abstraction to full mission deployment, integrating morphological, functional, and behavioral analogs into mechatronic subsystems. By explicitly linking biological principles with CAD-based modeling, hardware/software-in-the-loop validation, and ISO/VDI standards, Table A3 ensures methodological rigor, traceability, and reproducibility. In this sense, it serves as the technical backbone of the manuscript, demonstrating how biomimetic concepts evolve into space-qualified mechatronic systems.

**Table A3:** Analysis of steps included in BEAM-D standard.

BEAM-D	TRLs	Description
BEAM-D1 Space mission overview	TRL1	At this stage, the methodology anchors early discovery by systematically combining BIO1 (biological principle identification), M1 (mission requirements definition), BIO2 (biological data abstraction), and M2 (preliminary specifications). Together, these processes establish a robust foundation where biological inspiration is scientifically abstracted and aligned with initial space engineering requirements, ensuring that biomimetic analogs are selected with direct mission applicability in mind. The process begins with thoroughly analyzing the mission and applied space-environment parameters, forming the conceptual framework upon which all subsequent actions are scaffolded. User requirements are identified, encompassing desired functionality, performance benchmarks, and expected outcomes. This leads to the development of a conceptual solution strategy, including initial cost estimates, project timelines, and key milestones. Technical challenges are then identified and abstracted into core models, involving the dissection of complex issues into generalized conceptual patterns that expose systemic interdependencies and enable cross-domain exploration. The product concept is structured into functional modules with defined specifications, followed by the evaluation of available tools, limitations, and resources. Hardware components are selected, and software platforms, such as compilers and development platforms, are specified to enable multidisciplinary integration across mechanical, electrical, and software domains. Biomimetic knowledge underpins the formulation of emerging hardware concepts, software architectures, and mathematical models, culminating in detailed requirements and technical specification documents that guide the transition into subsequent steps of biomimetic mechatronic system development.
BEAM-D2: Space bionic robot development	TRL2	At this stage, the methodology anchors early discovery by systematically combining BIO1 (biological principle identification), M1 (mission requirements definition), BIO2 (biological data abstraction), and M2 (preliminary specifications). Together, these processes establish a robust foundation where biological inspiration is scientifically abstracted and aligned with initial space engineering requirements, ensuring that biomimetic analogs are selected with direct mission applicability in mind. The process begins with thoroughly analyzing the mission and applied space-environment parameters, forming the conceptual framework upon which all subsequent actions are scaffolded. User requirements are identified, encompassing desired functionality, performance benchmarks, and expected outcomes. This leads to the development of a conceptual solution strategy, including initial cost estimates, project timelines, and key milestones. Technical challenges are then identified and abstracted into core models, involving the dissection of complex issues into generalized conceptual patterns that expose systemic interdependencies and enable cross-domain exploration. The product concept is structured into functional modules with defined specifications, followed by the evaluation of available tools, limitations, and resources. Hardware components are selected, and software platforms, such as compilers and development platforms, are specified to enable multidisciplinary integration across mechanical, electrical, and software domains. Biomimetic knowledge underpins the formulation of emerging hardware concepts, software architectures, and mathematical models, culminating in detailed requirements and technical specification documents that guide the transition into subsequent steps of biomimetic mechatronic system development.
	TRL3	At this level, BIO4 (biomimetic translation), BIO5 (form-function coupling), and M4 (mathematical modeling and simulation) are integrated. This triad ensures that biological mechanisms are not only conceptually translated but also functionally embodied in experimental platforms, enabling laboratory validation under controlled conditions that confirm biomimetic feasibility. Mathematical models (kinematics and dynamics) for system components are developed using both theoretical and empirical approaches, applying modeling tools and identification techniques. These models provide accurate representations of physical processes and system behaviors, forming the foundation for analytical studies and laboratory demonstrations using the analog application found in Table A4—Appendix A. Simulations and limited-functionality prototypes using non-integrated software components are employed to confirm critical performance attributes and validate analytical predictions (CAE). In addition, in the biomimetic design process, practitioners engage in an iterative search and identification of biological models, refining their exploration trajectory with each cycle as new insights and terminology emerge. This cognitive loop enhances the precision of identified solution spaces and increases the likelihood of discovering functionally analogous biological locomotion or manipulation strategies.
	TRL4	It incorporates BIO6 (structural optimization of biological analogs), BIO7 (integration of multi-biological strategies), and the engineering stages M5 (component design), M6 (prototype development), and M7 (mechatronics components). These activities provide convergence between biological analog fidelity and space system engineering, resulting in integrated prototypes capable of operating in controlled laboratory environments while adhering to early VDI/ISO validation benchmarks. Mechatronics designs are finalized, followed by the integration of hardware and software into functional prototypes that prioritize system performance and human–machine interaction. Cyber-physical models are employed to evaluate interactions among mechanical, electrical, and software components, identify design flaws early, and support iterative refinement, thereby minimizing development risks. Full hardware assembly includes the integration of sensors, actuators, microcontrollers, electrical systems, cabling, switches, and communication buses into structural elements, ensuring readiness for system-level testing. A low-fidelity system/component breadboard is constructed and operated to demonstrate basic functionality in critical test environments, with performance predictions aligned to operational conditions. Key software elements are integrated and validated for interoperability, initiating architecture development and laying the groundwork for performance assessments. In parallel, the biomimetic process advances by extracting biological resolution elements, such as morphological patterns or functional mechanisms, from selected biological models, ensuring that only the essential, transferable insights are retained. These abstracted principles are then translated into technological design concepts through analogical reasoning and creative ideation, transforming biological strategies into innovative, enhanced engineering solutions that guide prototype development.
BEAM-D3: Space bionic robot testing	TRL5	At this intermediate maturity, BIO8 (robustness validation) is combined with M8 (hardware and software testing). These steps focus on scaling laboratory prototypes into field-relevant subsystems, tested under analog conditions that partially replicate extraterrestrial thermal, dust, and radiation stressors. Hardware-in-the-loop simulations are employed to replicate real-time system behavior, while software performance is evaluated under realistic operational conditions. Mechanical structures undergo stress and thermal analyses to ensure accuracy in response to environmental challenges and potential failure modes. A medium-fidelity system/component breadboard is constructed and operated within a simulated operational environment using realistic support elements to demonstrate performance in critical areas. End-to-end software components are implemented, interfaced with existing systems or simulations, and tested in representative environments to validate that system behavior meets predicted expectations. This step quantifies the prototype’s resilience and suitability for long-term deployment in safety-critical mechatronics applications, while also generating performance predictions for future development. Thus, the biomimetic process advances to implementation and validation through prototyping and testing, transitioning conceptual insights into tangible outputs. This involves a progression from virtual simulations to physical prototypes (CAM), which are subjected to rigorous evaluation within the original problem context, enabling refinement and optimization based on prior descriptive and analytical findings.
	TRL6	It extends BIO8 (robustness validation) into iterative engineering steps M9 (final hardware-system integration) and M10 (final software-system integration). Together, these ensure that integrated robotic subsystems demonstrate operational performance under complex analog conditions, verifying subsystem redundancy and reliability in preparation for high-fidelity field tests. The final system assembly incorporates mechanical and electrical adaptations to ensure functional and spatial compatibility among components, while manufacturing processes are optimized for cost-effectiveness and scalable production. Component synergies are exploited to enhance integration and reduce redundancy. Real sensor and actuator signals are used to calibrate system responses, with signal conditioning and filtering, improving data accuracy and enabling precise control parameter tuning under both steady and transient conditions. Human–machine interaction is validated to ensure usability and safety. A high-fidelity prototype that addresses all critical scaling challenges is built and tested within a relevant operational environment, confirming performance under demanding conditions. Software is implemented and assessed using full-scale, realistic problems, with partial integration into existing hardware/software systems, thereby demonstrating engineering feasibility and operational readiness. In parallel, the biomimetic design approach progresses through rigorous testing, transitioning from virtual simulations to physical models, uncovering critical process refinements and optimization strategies derived from earlier analytical steps.
	TRL7	At this advanced stage, BIO8 (final biological robustness iteration) is integrated with M11 (system testing) and M12 (mission scenario testing). This level confirms that biomimetic prototypes operate under highly representative space analog conditions (e.g., MDRS, volcanic fields, thermal-vacuum chambers), ensuring readiness for space qualification campaigns. The final system assembly incorporates mechanical and electrical adaptations to ensure functional and spatial compatibility among components, while manufacturing processes are optimized for cost-effectiveness and scalable production. Component synergies are exploited to enhance integration and reduce redundancy. Real sensor and actuator signals are used to calibrate system responses, with signal conditioning and filtering, improving data accuracy and enabling precise control parameter tuning under both steady and transient conditions. Human–machine interaction is validated to ensure usability and safety. A high-fidelity prototype that addresses all critical scaling challenges is built and tested within a relevant operational environment, confirming performance under demanding conditions. Software is implemented and assessed using full-scale, realistic problems, with partial integration into existing hardware/software systems, thereby demonstrating engineering feasibility and operational readiness. In parallel, the biomimetic design approach progresses through rigorous testing, transitioning from virtual simulations to physical models, uncovering critical process refinements and optimization strategies derived from earlier analytical steps.
	TRL8	Culminating, the M13 (production with qualification and certification to aerospace standards) validates the system for spaceflight. At this point, prototypes comply with standards by fulfilling VDI/ISO and aerospace certification requirements, confirming their readiness for orbital or planetary deployment. The final product is completed in its operational configuration and successfully validated through rigorous testing and analysis in the same analog environment of TRL7 or other [213]. Full functionality is demonstrated in simulated operational scenarios, with Verification and Validation (V&V) processes completed to confirm compliance and system readiness. Simultaneously, production infrastructure is established, involving the selection and configuration of manufacturing equipment, streamlined assembly line layouts, and robust quality control systems to ensure consistent, high-performance output. This marks the critical transition from engineering development to scalable manufacturing, ensuring the reliable production of flight-qualified space robotic systems prepared for large-scale deployment.
BEAM-D4: Space mission successfulness	TRL9	The space bionic robot is validated during real mission operations in the target extraterrestrial environment. The final product is successfully operated in its intended mission environment, demonstrating full functionality and performance under orbital or planetary conditions. All software is thoroughly debugged and fully integrated with operational hardware and software systems, and all user, maintenance, and training documentation is complete. Sustaining engineering support is established to ensure continued reliability and maintainability, confirming that the system is fully operational and proven through successful mission execution (Based on ISO 14619:2023).

The strategic importance of Table A4 lies in its role as the integrative bridge between conceptual methodology and applied validation within the BEAM-D framework. While the manuscript develops the theoretical and procedural dimensions of biomimetic standardization, Table A4 uniquely consolidates the set of simulators, virtual environments, and computational platforms that provide the experimental backbone for multi-domain verification. It demonstrates how each stage of the BEAM-D standard is digitally stress-tested prior to hardware development. This ensures methodological compliance with NASA’s TRL advancement, where simulator-based verification is a prerequisite for progressing from TRL 3 to TRL 5. Beyond TRL alignment, the table functions as a traceability matrix corresponding simulation domain, mechanical survivability, locomotion in reduced gravity, swarm coordination, or human–robot interaction. In space mechatronics, such explicit simulator documentation is not accessory but foundational: it guarantees reproducibility, cross-platform benchmarking, and international interoperability, aligning the BEAM-D approach with ISO/TC 266 and ISO 23835:2022 standards.

Therefore, the purpose to use the information from Table A4 in BEAM-D2 is to initiate the robot Development. Thus, BEAM-D2 is the phase for digital prototyping, control and perception algorithm development, co-simulation and SIL/HIL preparation, and the production of the digital twin that will drive physical component selection and bench validation. This step requires multiple simulation modalities: rigid-body/multi-body kinematics and dynamics, contact and collision, granular/terramechanics coupling, hydrodynamics, high-fidelity sensor emulation (LiDAR, RGBD, IMU, sonar), photorealistic rendering, and real-time execution for controller optimization. These are exactly the capabilities provided by the simulators listed in Table A4 and by their ecosystems (ROS [246], physics engines). BEAM-D2 is therefore the correct, and the appropriate step in our methodology to exercise these simulators comprehensively (SIL to HIL), prior to field validation. More critically, Table A4 transforms the manuscript from a methodological proposition into a digitally anchored, verifiable standardization framework, where every claim of feasibility is underpinned by a quantifiable simulation environment.

**Table A4:** Simulators for multi-environment robot applications during the BEAM-2 step.

		FEATURES																											
	SIMULATOR	Realistic Rendering	GPS	ROS Support	Tracks	Wheels	Legs	M/O wheels	Heightmap Import	OpenStreetMap	Path Planning	RGBD	LiDAR	Barometer	Sonar	Radar	ArduPilot	HIL	Virtual Reality	Hydrodynamics	Hydrostatics	Thruster	Fins	Pressure Sensor	DVL	C-Dyn	Wind	Waves	Water Currents
**Land** **Robots**	CoppeliaSim [247]	-	X	X	X	X	X	X	X	-	X	X	X	-	-	-	-	-	-	-	-	-	-	-	-	-	-	-	-
	Gazebo [248]	-	X	X	X	X	X	X	X	-	X	X	X	-	-	-	-	-	-	-	-	-	-	-	-	-	-	-	-
	RaiSim [249]	Unity	-	-	-	X	X	-	X	X	-	-	X	-	-	-	-	-	-	-	-	-	-	-	-	-	-	-	-
	Webots [250]	-	X	X	X	X	X	X	X	X	X	X	X	-	-	-	-	-	-	-	-	-	-	-	-	-	-	-	-
	PyBullet [251]	-	-	-	-	X	X	-	X	X	X	X	X	-	-	-	-	-	-	-	-	-	-	-	-	-	-	-	-
	CARLA [252]	Unreal	X	X	-	X	-	-	X	X	X	X	X	-	-	-	-	-	-	-	-	-	-	-	-	-	-	-	-
	Project Chrono [253]	Pov-ray	X	-	X	X	X	X	X	-	-	X	X	-	-	-	-	-	-	-	-	-	-	-	-	-	-	-	-
**Aerial** **Robots**	AirSim [254]	Unreal+ unity	X	X	-	-	-	-	-	-	-	X	X	X	-	-	X	X	X	-	-	-	-	-	-	-	-	-	-
	Flightmare [255]	Unity	-	X	-	-	-	-	-	-	-	X		-	-	-	-	-	X	-	-	-	-	-	-	-	-	-	-
	Gazebo [256]	-	X	X	-	-	-	-	-	-	-	X	X	X	X	X	X	X	X	-	-	-	-	-	-	-	-	-	-
	Webots [257]	-	X	X	-	-	-	-	-	-	-	X	X	-	X	X	X	-	X	-	-	-	-	-	-	-	-	-	-
**Underwater Robots**	UWSim-NET [258]	Osgocean	X	-	-	-	-	-	-	-	-	-	-	-	-	-	-	-	-	X	-	X	-	X	X	X	-	X	-
	UUV [259]	-	X	-	-	-	-	-	-	-	-	-	-	-	-	-	-	-	-	X	X	X	X	X	X	X	-	-	-
	URSim [260]	Unity	-	-	-	-	-	-	-	-	-	-	-	-	-	-	-	-	-	X	X	X	-	X	-	-	-	X	-
	USVSim [261]	-	X	-	-	-	-	-	-	-	-	-	-	-	-	-	-	-	-	X	X	X	X	X	X	X	X	X	X
	Stonefish [262]	Custom	X	-	-	-	-	-	-	-	-	-	-	-	-	-	-	-	-	X	X	X	-	X	X	X	X	X	X

Table A5 functions as a performance validation matrix, anticipating technological bottlenecks while aligning the design methodology with aerospace standards for reliability, maintainability, and long-term sustainability. Its significance is therefore twofold: first, it integrates biomimetic design logics with aerospace mechatronics engineering principles, ensuring structural and functional coherence across multiple domains; and second, it establishes a systematic roadmap for experimental validation and industrial translation, enabling BEAM-D to evolve from a conceptual biomimetic construct into a deployable architecture capable of supporting autonomous operations in deep space environments.

**Table A5:** Further considerations of BEAM-D implementation.

	State-of-the-Art Knowledge Gaps	Applied Biomimetic Innovative Benefits
**MANUFACTURING**	Current orbital manufacturing lacks the precision for variable stiffness, anisotropic layering, and real-time reconfigurability; unlike being supported by robots, these characteristics can achieve autonomous assembly. Materials and techniques fail to support multifunctional gradients or tunable rigidity [263]. Lunar manufacturing is further limited by regolith processing difficulties and the absence of autonomous, high-fidelity additive systems capable of producing structurally graded components under lunar dust, vacuum, and temperature fluctuations [264].	Using hybrid additive-subtractive techniques and programmable composites, it enables autonomous, mass-efficient assembly of structures mimicking organic growth. These materials self-adjust to thermal, radiative, and mechanical conditions. This design paradigm enhances the performance, durability, and adaptability of space robotics for exploration, construction, and long-duration planetary infrastructure deployment. Such robots dynamically reconfigure shape and function without external intervention [265]. Soft-rigid systems with tunable stiffness optimize interaction with delicate or volatile environments.
**TECHNICAL** **INTERDISCIPLINARITY**	Electromechanics, soft tissue analogs, and signal-processing matrices progress in isolation, slowing cohesive robotic development [266]. Planetary mobility benefits from bio-inspired movements, writhing, gripping, or embedding, that demand tight coupling of biomechanical schematics and adaptive control firmware. Disconnected methodologies limit rover evolution, delaying tools designed for volatile terrain interaction, cryogenic excavation, or particulate-resistant traversal on moons, comets, or ringed-body surfaces with unstable substrates [267].	Incorporating structured mechanisms for bridging divergent disciplinary approaches. For instance, in the case of robotic manipulation systems, BEAM-D facilitates the integration of aerospace-grade actuation and control architectures with biomimetic soft-tissue compliance models inspired by cephalopod muscular hydrostats, thereby enabling manipulators that combine high precision with adaptive flexibility in microgravity [268]. Similarly, the methodology enables the fusion of insect-inspired multimodal sensor arrays with space-qualified communication buses and embedded diagnostics, ensuring robust perception under extreme signal-to-noise conditions.
**EVOLUTIONARY BIOLOGY**	Simulating nature-driven electromechanical behaviors remains imprecise. Shape prediction engines, probabilistic mapping frameworks, and morphodynamics solvers fall short when designing metamorphic systems [269]. Interplanetary machines mimicking serpentine or insect-like propulsion must account for step change kinetics, sensor-actuator lag, and composite interaction rules. Incomplete digital ecosystems restrict application in scout fabrication, terrain-tracking devices, or autonomous reconfigurable swarms for exploratory or maintenance objectives [270].	Natural refinement acts as a biological algorithm, continuously improving traits for survival. This adaptive pathway informs machine configurations with fault tolerance, minimal energy demand, and responsive behavior. Translating phylogenetic success into mechanical morphology advances the development of robust crawlers, aerial scouts, and station utilities, ensuring reliable function in unstructured landscapes and under intermittent power availability during interplanetary operations [271]. Also, it enhances redundancy management in modular electromechanical systems for fault isolation and recovery.

**Dataset A. Comparative performance metrics of robotic systems from terrestrial analog testing**

Regarding sensors and sampling, wheel encoder counts and commanded wheel velocities (1–10 Hz), high-rate IMU (≥100 Hz) fused to compute slip; LiDAR scans (10–20 Hz) for mapping and SLAM validation; on-board current/voltage sampling (≥1 Hz) for energy accounting; temperature probes (1 Hz) on batteries, motors and electronics. Where possible, ground truth survey (RTK-GNSS [1]) is used to compute absolute positional error for SLAM. All reported numbers are mean ± standard error with sample size n indicated; recommended n ≥ 10 independent traverses or drillholes to produce robust statistics in field conditions [2]. The following data presents comparative results from the analysis of robots tested in analog sites:

(1) Basaltic volcanic terrains (Mauna Loa/Kīlauea):
  - Terrain properties: coarse basaltic grains, blocky substrates and variable fines; used extensively for rover trafficability and science tests in Hawaii analog campaigns (Mauna Kea/Mauna Loa field trials). Representative regolith analog characterization and rover instrumented traverses are reported. Typical mobility observations in the literature: sinkage s ≈ 4–6 cm for small/medium rovers under 20–30 kg mass and wheel diameters 0.15–0.35 m; rolling resistance coefficients measured C_rr ≈ 0.04–0.06; slip ratios commonly 10–25% on steep or blocky patches depending on wheel geometry. These numbers agree with terramechanics DEM validations for rough basaltic terrain. Designers should therefore target torque margins ≥1.2–1.5× nominal model predictions for basaltic analogs [3].
  - Energy example (25 kg rover at v = 0.10 m·s^−1^): with C_rr range 0.04–0.06, drive power P_drive = 0.981–1.471 W (drive-only); E_m = 9.807–14.710 J·m^−1^. In practice, total system power to traverse includes (1) actuator inefficiencies (assume η_drive ≈ 0.75–0.85), (2) electronic loads, and (3) intermittent thermal loads that produce operational energy budgets closer to tens of watts depending on duty cycles, these additional items should be added explicitly in mission energy budgets [2].
(2) Mars Desert Research Station (MDRS), Utah (fine iron-rich sediments):
  - Terrain properties: iron-rich fine particles, silt/sand fractions with median grain size often 30–150 µm in Utah/MDRS study areas; MDRS is used routinely to validate autonomy and human–robot interaction studies and to stress dust mitigation strategies. Field studies at MDRS and similar Utah analogs report higher sinkage and rolling resistance than basaltic lava flows, with representative C_rr ≈ 0.09–0.12, slip ratios frequently observed in the 15–30% range during loose fine-grained traverses, and sinkage s ≈ 6–9 cm for the same wheel/vehicle configurations cited above. These empirical findings are supported by LPI/MDRS rover route mapping and field mobility experiments [4].
  - Energy example (25 kg rover at v = 0.10 m·s^−1^): with C_rr 0.09–0.12, drive power P_drive = 2.2065–2.9420 W (drive-only); E_m = 22.065–29.420 J·m^−1^. Compared to basaltic terrains this implies an increase in drive energy of ≈ 2.25–3.0× (drive-only) when the vehicle moves from low-C_rr lava flats to MDRS fine-particle terrain (note: this ratio is for rolling resistance only; the field total energy penalty often reaches ≈20–40% when added thermal, sensing and recovery actions are included). These values agree with DEM and experimental wheel-soil studies that show energy and torque increase with decreasing grain size and increased sinkage [5].
  - SLAM and sensing: LiDAR SLAM map overlap (IoU with ground truth point cloud) [6] in Utah/MDRS field runs typically lies in the 89–93% range using high-rate LiDAR with offline registration (reported values vary by sensor suite and environmental occlusion); drift rates of 0.3–0.6% per 100 m are commonly reported in comparable field trials when wheel odometry is fused with IMU and LiDAR constraints. These SLAM performance ranges are consistent with the SLAM benchmarking literature comparisons. Reported autonomy waypoint success rates in MDRS trials are often 80–95% depending on mission complexity and dust contamination [7].
  - Dust and contamination: fine particulates increase optical sensor noise, degrade solar arrays and cause particulate fouling on mechanical contacts; empirical Mars rover studies and terrestrial analog field campaigns show optical signal-to-noise ratio (SNR) reductions of 10–20% between clean and dust-exposed conditions and measurable solar panel power losses if dust deposition is not mitigated. These practical observations motivate sealed bearings, filtered vents, dust-tolerant encoders and active optical self-cleaning cycles for BEAM-D3 prototypes prior to TRL advancement [8].
(3) High-altitude analogs (>4000 m, Andes and similar):
  - Environmental effects: reduced ambient pressure (~0.6 bar at ~4000 m) lowers convective heat transfer coefficient h, which in turn reduces passive cooling capacity of enclosures and increases equilibrium internal temperatures for the same internal dissipation; multiple experimental studies of electronics cooling show convective heat transfer coefficient decreases and internal temperature rises by 8–14 °C for typical forced-air designs unless forced-air mass flow is increased. UV irradiance also increases with altitude (empirical values: ≈5–12% increase per 1000 m depending on aerosol/ozone conditions), both phenomena affect materials aging, optics degradation and thermal design choices. Designers must therefore validate thermal control (fin area, forced-air, or active cooling) at altitude or incorporate margin for increased active cooling power [9].
  - Quantitative guidance: for electronics dissipation of 30–100 W in a small rover chassis tested at sea level, designers may observe an internal ΔT increase of ~10 °C at 4000 m with identical heat-sink geometry and fan speeds (empirical value from electronics cooling experiments). To maintain sea-level thermal margins, increase forced-air mass flow or radiator area, or plan for active cooling, quantify by performing a STOP analysis (structural-thermal-optical) campaign for optics and thermal loop calculations for electronics [10].
(4) Antarctic/polar and under-ice analogs:
  - For robotics targeted at icy worlds or polar science, Antarctic trials show that mechanical traction, thermal management, and power provisioning present distinct operational envelopes (low temperatures, ice adhesion/abrasion, and very low sunlight months). Australian Antarctic Program field tests of under-ice and polar robots (e.g., BRUIE [11], IceNode [12]) demonstrate the need for specialized seals, low-temperature batteries or heaters and autonomous fault-recovery due to limited field servicing [13]. When comparing polar to desert analogs, they expect substantially higher heater energy fractions and different wheel/track traction behavior; designers must therefore treat polar trials as validating thermal and seals rather than typical regolith traction.

## References

[1] Nisbet E., Sleep N. (2001). The habitat and nature of early life. Nature.

[2] Goel A.K., McAdams D.A., Stone R.B. (2013). Biologically Inspired Design: Computational Methods and Tools, 2014 ed..

[3] Nachtigall W. (2010). Bionik als Wissenschaft: Erkennen-Abstrahieren-Umsetzen.

[4] Barrera A., Cáceres A., Weitzenfeld A., Ramirez-Amaya V. (2011). Comparative experimental studies on spatial memory and learning in rats and robots. J. Intell. Robot. Syst..

[5] Benyus J.M. (1997). Biomimicry: Innovation Inspired by Nature.

[6] Montana Hoyos C., Fiorentino C. (2016). Bio-utilization, bio-inspiration, and bio-affiliation in design for sustainability: Biotechnology, biomimicry, and biophilic design. Int. J. Des. Objects.

[7] Prólogo. Arruda A., Langella C. (2021). Prólogo. BioDiseño, Innovación y Transdisciplinariedad. Cuad. Cent. Estud. Diseño Comun. Ens..

[8] Stone R.B., Goel A.K., McAdams D.A. (2013). Charting a course for computer-aided bio-inspired design. Biologically Inspired Design: Computational Methods and Tools.

[9] Wanieck K., Beismann H. (2021). Perception and role of standards in the world of biomimetics. Bioinspired Biomim. Nanobiomaterials.

[10] Speck O., Speck D., Horn R., Gantner J., Sedlbauer K.P. (2017). Biomimetic bio-inspired biomorph sustainable? An attempt to classify and clarify biology-derived technical developments. Bioinspiration Biomim..

[11] Datteri E., Tamburrini G. (2007). Biorobotic Experiments for the Discovery of Biological Mechanisms. Philos. Sci..

[12] Graeff E., Letard A., Raskin K., Maranzana N., Aoussat A. (2021). Biomimetics from practical feedback to an interdisciplinary process. Res. Eng. Des..

[13] Weitzenfeld A., Vallesa A., Flores H. A biologically-inspired wolf pack multiple robot hunting model. Proceedings of the 2006 IEEE 3rd Latin American Robotics Symposium.

[14] Siddall R., Zufferey R., Armanini S., Zhang K., Sareh S., Sergeev E. (2023). The natural robotics contest: Crowdsourced biomimetic design. Bioinspiration Biomim..

[15] (2025). Biomimetics.

[16] Wanieck K. (2022). Biomimetics for Technical Products and Innovation.

[17] Bar-Cohen Y. (2016). Biomimetics: Nature-Based Innovation.

[18] Jiang Z., Ma Y., Xiong Y. (2023). Bio-inspired generative design for engineering products: A case study for flapping wing shape exploration. Adv. Eng. Inform..

[19] Fattepur G., Patil A.Y., Kumar P., Kumar A., Hegde C., Siddhalingeshwar I.G., Kumar R., Khan T.M.Y. (2024). Bio-inspired designs: Leveraging biological brilliance in mechanical engineering—An overview. 3 Biotech.

[20] De Pauw I., Kandachar P., Karana E. (2015). Assessing sustainability in nature-inspired design. Int. J. Sustain. Eng..

[21] Bhushan B. (2009). Biomimetics: Lessons from nature–an overview. Philos. Trans. R. Soc. A Math. Phys. Eng. Sci..

[22] Yen J., Weissburg M. (2007). Perspectives on biologically inspired design: Introduction to the collected contributions. Bioinspiration Biomim..

[23] Zhang J., Kestem L., Wommer K., Wanieck K. (2025). Biomimetic tools: Insights and implications of a comprehensive analysis and classification. Bioinspiration Biomim..

[24] Kiakojouri F., De Biagi V., Abbracciavento L. (2023). Design for robustness: Bio-inspired perspectives in structural engineering. Biomimetics.

[25] Gul F., Mir I., Abualigah L., Sumari P. (2021). Multi-robot space exploration: An augmented arithmetic approach. IEEE Access.

[26] Bradley D. (2010). Mechatronics–More questions than answers. Mechatronics.

[27] Kyura N., Oho H. (1996). Mechatronics-an industrial perspective. IEEE/ASME Trans. Mechatron..

[28] Zheng C., Bricogne M., Le Duigou J., Eynard B. (2014). Survey on mechatronic engineering: A focus on design methods and product models. Adv. Eng. Inform..

[29] Dixit U.S., Hazarika M., Davim J.P. (2017). A Brief History of Mechanical Engineering.

[30] Vazquez-Santacruz J.A., Portillo-Velez R., Torres-Figueroa J., Marin-Urias L., Portilla-Flores E. (2023). Towards an integrated design methodology for mechatronic systems. Res. Eng. Des..

[31] Isermann R. (1996). Modeling and design methodology for mechatronic systems. IEEE/ASME Trans. Mechatron..

[32] Isermann R. (2023). Designs and Specification of Mechatronic Systems. Springer Handbook of Automation.

[33] Hehenberger P., Bradley D. (2016). Mechatronic Futures. Challenges and Solutions for Mechatronic Systems and Their Designers.

[34] Bradley D., Russell D., Ferguson I., Isaacs J., MacLeod A., White R. (2015). The Internet of Things–The future or the end of mechatronics. Mechatronics.

[35] Masior J., Schneider B., Kürümlüoglu M., Riedel O. (2020). Beyond model-based systems engineering towards managing complexity. Procedia CIRP.

[36] Chen R., Liu Y., Zhao J., Ye X. (2019). Model verification for system design of complex mechatronic products. Syst. Eng..

[37] Lettner D., Hehenberger P., Nöhrer A., Anzengruber K., Grünbacher P., Mayrhofer M., Egyed A. (2015). Variability and consistency in mechatronic design. Concurr. Eng..

[38] Egyed A., Zeman K., Hehenberger P., Demuth A. (2018). Maintaining consistency across engineering artifacts. Computer.

[39] Borchani M.F., Ammar R., Hammadi M., Choley J.-Y., Yahia N.B., Barkallah M., Louati J. Mechatronic system design using model-based systems engineering and set-based concurrent engineering principles. Proceedings of the 2018 12th France-Japan and 10th Europe-Asia Congress on Mechatronics.

[40] Ellery A. (2020). Tutorial review of bio-inspired approaches to robotic manipulation for space debris salvage. Biomimetics.

[41] Menon C., Broschart M., Lan N. Biomimetics and robotics for space applications: Challenges and emerging technologies. Proceedings of the IEEE International Conference on Robotics and Automation-Workshop on Biomimetic Robotics.

[42] Ayre M. (2004). Biomimetics applied to space exploration. WIT Trans. Ecol. Environ..

[43] Hall L. NASA Innovative Advanced Concepts. https://www.nasa.gov/stmd-the-nasa-innovative-advanced-concepts-niac/.

[44] ESA Advanced Concepts Teams. https://www.esa.int/gsp/ACT/.

[45] Le C.L., Yirmibesoglu O.D., Even S., Buckner T., Ozkan-Aydin Y., Kramer-Bottiglio R. (2025). Grand challenges for burrowing soft robots. Front. Robot. AI.

[46] Cornejo J., García Cena C.E., Baca J. (2024). Animal-Morphing Bio-Inspired Mechatronic Systems: Research Framework in Robot Design to Enhance Interplanetary Exploration on the Moon. Biomimetics.

[47] Weitzenfeld A., Arbib M.A., Alexander A. (2002). The Neural Simulation Language: A System for Brain Modeling.

[48] Banken E., Oeffner J. (2023). Biomimetics for innovative and future-oriented space applications-A review. Front. Space Technol..

[49] Miller S.K., Banks B.A. Atomic Oxygen Environments, Effects, and Mitigation. Proceedings of the Applied Space Environments Conference (ASEC) 2019.

[50] Siochi E.J., Anders Jr J.B., Cox D.E., Jegley D.C., Fox R.L., Katzberg S.J. (2002). Biomimetics for NASA Langley Research Center: Year 2000 Report of Findings from a Six-Month Survey.

[51] Finckenor M.M., de Groh K. (2017). A researcher’s guide to: Space environmental effects. Res. Guide Ser. Natl. Aeronaut. Spac E Adm. Int. Space Stn..

[52] Domínguez R., Pérez-del-Pulgar C., Paz-Delgado G.J., Polisano F., Babel J., Germa T., Dragomir I., Ciarletti V., Berthet A.-C., Danter L.C. (2025). Cooperative robotic exploration of a planetary skylight surface and lava cave. Sci. Robot..

[53] Egan P., Sinko R., LeDuc P.R., Keten S. (2015). The role of mechanics in biological and bio-inspired systems. Nat. Commun..

[54] O’Connor S., Plecnik M. (2025). The Synthesis of Spherical Four-Bars for Biomimetic Motion Through Complete Solutions for Approximate Rigid Body Guidance. J. Mech. Robot..

[55] Pernigoni L., Lafont U., Grande A.M. (2021). Self-healing materials for space applications: Overview of present development and major limitations. CEAS Space J..

[56] Evans B. (2022). NASA’s Voyager Missions: Exploring the Outer Solar System and Beyond.

[57] Ellery A. Bioinspiration lessons from a self-replicating machine concept in a constrained environment. Proceedings of the 2017 IEEE International Conference on Robotics and Biomimetics (ROBIO).

[58] Scott G.P., Ellery A. (2004). Biomimicry as Applied to Space Robotics. https://d1wqtxts1xzle7.cloudfront.net/75278646/Biomimicry_as_Applied_to_Space_Robotics_20211127-16304-1q2k5xy.pdf?1738451568=&response-content-disposition=inline%3B+filename%3DBiomimicry_as_Applied_to_Space_Robotics.pdf&Expires=1759499692&Signature=LRFGL6qtjtsYzziOYjSQnFL6siRPikgjFN9INGbxUUgyI7DxYtHvVAa~0nRVfmfx48hH1q8f6e4WOr1g0dVAyr~t5t5jggKMniXMwwtJ4n5bqqvevwTpkNTAg8tw3JbOTtdW~PpQIAlq~XPhBPwZg-It~wUOCtfegYjIXZFkJxP0mYAFpBQPvizyH9GVXUfiYJOyqicbAggn5n9AxDrV0lvsfrbbA9rL7JLK0JFU3m6xWlUWL-6PFOpbaTssS2bKRwqsoq459jbXeipddOdzWkMgMLiLE8QLX8VkfRPATjVUaypIbgt6WqbQkgbx9xNH7P4lFCk1wz03ltkw3-XogA__&Key-Pair-Id=APKAJLOHF5GGSLRBV4ZA.

[59] Helms M., Vattam S.S., Goel A.K. (2009). Biologically inspired design: Process and products. Des. Stud..

[60] Helten K., Schenkl S., Lindemann U. Biologizing product development—Results from a student project. Proceedings of the ICORD 11: Proceedings of the 3rd International Conference on Research into Design Engineering.

[61] Graeff E., Maranzana N., Aoussat A. (2019). Engineers’ and biologists’ roles during biomimetic design processes, towards a methodological symbiosis. Proc. Des. Soc. Int. Conf. Eng. Des..

[62] Vattam S.S., Helms M.E., Goel A.K. (2010). A content account of creative analogies in biologically inspired design. Artif. Intell. Eng. Des. Anal. Manuf..

[63] Graeff E., Maranzana N., Aoussat A. (2019). Biomimetics, where are the biologists?. J. Eng. Des..

[64] Ngo T.D. (2015). Biomimetic Technologies: Principles and Applications.

[65] Arkin R.C., Ali K., Weitzenfeld A., Cervantes-Pérez F. (2000). Behavioral models of the praying mantis as a basis for robotic behavior. Robot. Auton. Syst..

[66] Cohen Y.H., Reich Y. (2016). Biomimetic Design Method for Innovation and Sustainability.

[67] Clarkson J., Eckert C. (2005). Design Process Improvement: A review of current practice.

[68] Fayemi P.E., Wanieck K., Zollfrank C., Maranzana N., Aoussat A. (2017). Biomimetics: Process, tools and practice. Bioinspiration Biomim..

[69] Altshuller G.S. (1984). Creativity as an Exact Science.

[70] Hoagland M. (1995). The Way Life Works.

[71] Tinsley A., Midha P.A., Nagel R.L., McAdams D.A., Stone R.B., Shu L.H. Exploring the use of functional models as a foundation for biomimetic conceptual design. Proceedings of the International Design Engineering Technical Conferences and Computers and Information in Engineering Conference.

[72] VDI (2025). VDI manual Biomimetics. https://www.vdi.de/en/home/vdi-standards/details/vdi-manual-biomimetics.

[73] Sartori J., Pal U., Chakrabarti A. (2010). A methodology for supporting “transfer” in biomimetic design. Artif. Intell. Eng. Des. Anal. Manuf..

[74] (2023). Part 2—Biomimetics—Biomimetic Design Methodology—Products and Processes.

[75] (2021). Part 1—Biomimetics—Fundamentals, Conception, and Strategy.

[76] (2017). Part 3—Biomimetics—Integrated Product Development Process for Biomimetic Optimisation.

[77] (2016). Biomimetics—Biomimetic Materials, Structures and Components.

[78] (2015). Biomimetics—Terminology, Concepts and Methodology.

[79] (2015). Biomimetics—Biomimetic Structural Optimization.

[80] (2015). Part 1—Biomimetics—Architecture, Civil Engineering, Industrial Design—Basic Principles.

[81] (2013). Part 1—Biomimetics—Biomimetic Robots.

[82] (2013). Part 1—biomimetics—Biomimetic Surfaces.

[83] (2013). Part 1—Biomimetics—Biomimetic Materials, Structures and Components.

[84] (2012). Part 1—Biomimetics—Biomimetic Information Processing.

[85] (2012). Part 2—Biomimetic Optimization—Application of Biological Growth Laws for the Structure-Mechanical Optimization of Technical Components.

[86] (2012). Part 1—Biomimetic Optimization—Application of Evolutionary Algorithms.

[87] (2011). Biomimetics—Conception and Strategy—Differences Between Bionic and Conventional Methods/Products.

[88] Fayemi P. (2016). Innovation Through Bio-Inspired Design: Suggestion of a Structuring Model for Biomimetic Process and Methods.

[89] Gentner D. (1989). Analogical Learning.

[90] Weidner B.V., Nagel J., Weber H.-J. (2018). Facilitation method for the translation of biological systems to technical design solutions. Int. J. Des. Creat. Innov..

[91] Bolton W. (2017). Mecatrónica.

[92] Chhabra R., Emami M.R. (2014). A holistic approach to concurrent engineering and its application to robotics. Concurr. Eng..

[93] Pahl G., Beitz W., Feldhusen J., Grote K.H. (2007). Engineering Design: A Systematic Approach.

[94] van Amerongen J. (2004). Mechatronics Education and Research–15 Years of Experience. IFAC Proc. Vol..

[95] Dixit U.S., Hazarika M., Davim J.P. (2017). History of mechatronics. A Brief History of Mechanical Engineering.

[96] Craig J.J. (2006). Robótica.

[97] Yang C., Ma H., Fu M. (2016). Robot kinematics and dynamics modeling. Advanced Technologies in Modern Robotic Applications.

[98] Anciferov S.I., Gaponenko E.V., Kuzmina V.S. (2019). Robotic system development using CAD/CAM/CAE of NX. J. Phys. Conf. Ser..

[99] van Wynsberghe A. (2013). A method for integrating ethics into the design of robots. Ind. Robot Int. J..

[100] Jamaludin J., Rohani J.M. Cyber-physical system (cps): State of the art. Proceedings of the 2018 international conference on computing, electronic and electrical engineering (ICE cube).

[101] (2021). Development of Mechatronic and Cyber-Physical Systems.

[102] Gausemeier J., Moehringer S. New guideline vdi 2206-a flexible procedure model for the design of mechatronic systems. Proceedings of the ICED 03, the 14th International Conference on Engineering Design.

[103] STARTS G. (1989). The STARTS Purchases Handbook: Software Tools for Application to Large Real-Time Systems.

[104] Graessler I., Hentze J. (2020). The new V-Model of VDI 2206 and its validation. at-Autom..

[105] Mourtzis D., Angelopoulos J., Panopoulos N. (2023). The future of the human–machine interface (HMI) in society 5.0. Future Internet.

[106] Daiker R., Ghatas R., Vincent M., Rippy L., Holbrook J. (2019). A Cognitive Task Analysis of Safety-Critical Launch Termination Systems. Advances in Human Aspects of Transportation.

[107] ISO (2015). ISO/TC 299 Robotics. https://www.iso.org/committee/5915511.html.

[108] (2025). Robotics—Safety Requirements Part 1: Industrial robots.

[109] (2025). Robotics—Safety Requirements Part 2: Industrial robot applications and robot cells.

[110] (2016). Robots and Robotic Devices—Collaborative robots.

[111] (2023). Robotics—Collaborative Applications—Test Methods for Measuring Forces and Pressures in Human-Robot Contacts.

[112] (2024). Robotics—Vocabulary.

[113] (1995). Manipulating Industrial Robots—Informative Guide on Test Equipment and Metrology Methods of Operation for Robot Performance Evaluation in Accordance with ISO 9283.

[114] (1998). Manipulating Industrial Robots—Performance Criteria and Related Test Methods.

[115] (1999). Manipulating Industrial Robots—Presentation of Characteristics.

[116] (2000). Manipulating Industrial Robots—Object Handling with Grasp-Type Grippers—Vocabulary and Presentation of Characteristics.

[117] (2002). Manipulating Industrial Robots—Mechanical Interfaces.

[118] (2004). Manipulating Industrial Robots—Mechanical Interfaces.

[119] (2013). Robots and Robotic Devices—Coordinate Systems and Motion Nomenclatures.

[120] (2022). Robots for Industrial Environments—Automatic end Effector Exchange Systems—Vocabulary.

[121] (2014). Robotics—Safety Requirements for Service Robots.

[122] (2016). Robotics—Performance Criteria and Related Test Methods for Service Robots, Part 1: Locomotion for Wheeled Robots.

[123] (2021). Robotics—Performance Criteria and Related Test Methods for Service Robots, Part 3: Manipulation.

[124] (2021). Robotics—Performance Criteria and Related Test Methods for Service Robots, Part 4: Lower-Back Support Robots.

[125] (2024). Robotics—Performance Criteria and Related Test Methods for Service Robots, Part 2: Navigation.

[126] (2025). Robotics—Performance Criteria and Related Test Methods for Service Robots, Part 5: Locomotion for Legged Robots.

[127] (2025). Robotics—Performance Criteria and Related Test Methods for Service Robots, Part 6: Lower-Limb Wearable Robots.

[128] (2025). Robotics—Performance Criteria and Related Test Methods for Service Robots, Part 7: Travelling Around Humans.

[129] (2025). Robotics—Performance Criteria and Related Test Methods for Service Robots, Part 8: Electric Vehicle Charging Robots.

[130] (2017). Mobile Robots—Vocabulary.

[131] (2025). Robotics—Autonomous Mobile Robots for Industrial Environments—Communications and Interoperability.

[132] (2021). Robotics—Modularity for Service Robots Part 1: General Requirements.

[133] (2024). Robotics—Modularity for Service Robots Part 201: Common Information Model for Modules.

[134] (2024). Robotics—Modularity for Service Robots Part 203: Information Model for Hardware.

[135] (2025). Robotics—Modularity for Service Robots Part 202: INFORMATION Model for Software Modules.

[136] (2025). Robotics—Test Methods for Measuring the Energy Consumption of Robots—6-Axis Articulated Industrial Robots.

[137] Cermak A. (2023). Chapter 9: Spacecraft Classification. https://science.nasa.gov/learn/basics-of-space-flight/chapter9-1/.

[138] Leonard J., Nornholm R. The process of applying automation and robotics standards in space systems. Proceedings of the Space Programs and Technologies Conference and Exhibit.

[139] Weisbin C.R., Lavery D.B., Rodriguez G. (1997). Robots in space: US missions and technology requirements into the next century. Auton. Robot..

[140] Visentin G., Van Winnendael M., Putz P. Advanced mechatronics in ESA’s space robotics developments. Proceedings of the 2001 IEEE/ASME International Conference on Advanced Intelligent Mechatronics. Proceedings (Cat. No. 01TH8556).

[141] Hirzinger G., Sporer N., Schedl M., Butterfass J., Grebenstein M. Robotics and mechatronics in aerospace. Proceedings of the 7th International Workshop on Advanced Motion Control. Proceedings (Cat. No. 02TH8623).

[142] Wang Z., Wang P., Duan J., Tian W. (2025). Review of On-Orbit Assembly Technology with Space Robots. Aerospace.

[143] Kramer G. (2022). Origin, Geography, and Geology of the Moon. Handbook of Lunar Base Design and Development.

[144] Daubar I.J., Dundas C.M., McEwen A.S., Gao A., Wexler D., Piqueux S., Collins G.S., Miljkovic K., Neidhart T., Eschenfelder J. (2022). New craters on Mars: An updated catalog. J. Geophys. Res. Planets.

[145] Coloma S., Martinez C., Yalçın B.C., Olivares-Mendez M.A. (2022). Enhancing rover teleoperation on the moon with proprioceptive sensors and machine learning techniques. IEEE Robot. Autom. Lett..

[146] Cornell Law S. (2022). 50 U.S. Code § 3373-Establishment of All-Domain Anomaly Resolution Office. https://www.law.cornell.edu/uscode/text/50/3373.

[147] Tang Q., Liang J., Zhu F. (2023). A comparative review on multi-modal sensors fusion based on deep learning. Signal Process..

[148] IEEE (2025). Space Robotics-IEEE Robotics and Automation Society (RAS). https://www.ieee-ras.org/space-robotics.

[149] (2015). IEEE Standard Ontologies for Robotics and Automation.

[150] (2021). IEEE Standard for Autonomous Robotics (AuR) Ontology.

[151] Smith C., Mott T., Williams T. Robot, Take the Joystick: Understanding Space Robotics Experts’ Views on Autonomy. Proceedings of the 2024 33rd IEEE International Conference on Robot and Human Interactive Communication (ROMAN).

[152] Bavle H., Sanchez-Lopez J.L., Cimarelli C., Tourani A., Voos H. (2023). From slam to situational awareness: Challenges and survey. Sensors.

[153] Bernardo R., Sousa J.M., Gonçalves P.J. (2023). A novel framework to improve motion planning of robotic systems through semantic knowledge-based reasoning. Comput. Ind. Eng..

[154] (2015). IEEE Standard for Robot Map Data Representation for Navigation.

[155] (2025). IEEE Draft Standard for 3D Map Data Representation for Robotics and Automation.

[156] Alghamdi S., Alahmari S., Yonbawi S., Alsaleem K., Ateeq F., Almushir F. Autonomous Navigation Systems in GPS-Denied Environments: A Review of Techniques and Applications. Proceedings of the 2025 11th International Conference on Automation, Robotics, and Applications (ICARA).

[157] Nguyen L.A., Harman T.L., Fairchild C. Swarmathon: A swarm robotics experiment for future space exploration. Proceedings of the 2019 IEEE International Symposium on Measurement and Control in Robotics (ISMCR).

[158] Shyam V., Friend L., Whiteaker B., Bense N., Dowdall J., Boktor B., Johny M., Reyes I., Naser A., Sakhamuri N. (2019). PeTaL (Periodic Table of Life) and Physiomimetics. Designs.

[159] Wood D., Rathnasabapathy M., Stober K.J., Menon P. (2024). Challenges and progress in applying space technology in support of the sustainable development goals. Acta Astronaut..

[160] Haidegger T., Mai V., Mörch C.M., Boesl D.O., Jacobs A., Rao R.B., Khamis A., Lach L., Vanderborght B. (2023). Robotics: Enabler and inhibitor of the sustainable development goals. Sustain. Prod. Consum..

[161] Haley A.G. (1955). International cooperation in rocketry and astronautics. J. Jet. Propuls..

[162] (2022). Space Systems—Mechanism Design and Verification.

[163] (2023). Space Systems—Space Experiments—General Requirements.

[164] NASA (2020). Technology Readiness Levels (TRL). https://www.nasa.gov/directorates/somd/space-communications-navigation-program/technology-readiness-levels/.

[165] Alkalla M., Pang X., Pitcher C., Gao Y. (2021). DROD: A hybrid biomimetic undulatory and reciprocatory drill: Quantitative analysis and numerical study. Acta Astronaut..

[166] Pitcher C., Alkalla M., Pang X., Gao Y. (2020). Development of the third generation of the dual-reciprocating drill. Biomimetics.

[167] Cfdem (2020). LIGGGHTS® Open Source Discrete Element Method Particle Simulation Code. https://www.cfdem.com/liggghtsr-open-source-discrete-element-method-particle-simulation-code.

[168] Alkalla M., Pitcher C. (2023). DROD: Bio-robotic drill/sampler for planetary subterranean exploration: Experiments and challenges. Bioinspiration Biomim..

[169] Khanna N., Prajapati R., Badheka V., Fuse K., Singh M., Palanisamy S. In Pursuit of Achieving High-Speed Drilling of 3d-Printed Inconel-718 Under Extreme Condition. https://ssrn.com/abstract=5394736.

[170] Zacny K., Bar-Cohen Y., Brennan M., Briggs G., Cooper G., Davis K., Dolgin B., Glaser D., Glass B., Gorevan S. (2008). Drilling systems for extraterrestrial subsurface exploration. Astrobiology.

[171] Barker D.C., Olivas A., Farr B., Wang X., Buhler C.R., Wilson J., Mai J. (2022). Adhesion of lunar simulant dust to materials under simulated lunar environment conditions. Acta Astronaut..

[172] Costa N., Bonetto A., Ferretti P., Casarotto B., Massironi M., Altieri F., Nava J., Favero M. (2024). Analytical data on three Martian simulants. Data Brief..

[173] NASA (2025). Science Misions. https://science.nasa.gov/science-missions/.

[174] Pitcher C., Pang X., Alkalla M., Gao Y. (2022). Development of a multi-sample acquisition technique for efficient planetary subsurface exploration. Acta Astronaut..

[175] Pitcher C., Alkalla M., Pang X., Yang Gao X. (2021). Biologically-inspired mechanisms for space applications. Space Robotics and Autonomous Systems: Technologies, Advances and Applications.

[176] Yuan W., Zhang C., Ling Z., Zhao D., Wang W., Chen Y., Lu F., Zhang S.-N., Cui W. (2018). Einstein Probe: A Lobster-Eye Telescope for Monitoring the X-Ray Sky. https://www.spiedigitallibrary.org/conference-proceedings-of-spie/10699/2313358/Einstein-Probe--a-lobster-eye-telescope-for-monitoring-the/10.1117/12.2313358.short.

[177] Peng S., Wei F., Guo Y., Ye Y. (2019). Preliminary geometric parameters optimization of lobster-eye-type wide field of view soft x-ray imager. Opt. Eng..

[178] Nurhaniza M., Ariffin M.K.A., Ali A., Mustapha F., Noraini A.W. (2010). Finite element analysis of composites materials for aerospace applications. IOP Conf. Ser. Mater. Sci. Eng..

[179] Li L., Zhang C., Jin G., Yuan W., Zhang S., Li Z., Gu Y., Wang J., Zhang Z., Zhang Z. (2021). Study on the optical properties of Angel Lobster eye X-ray flat micro pore optical device. Opt. Commun..

[180] Richter M.H., Cheng W.-H., Crumlin E.J., Drisdell W.S., Atwater H.A., Schmeißer D., Lewis N.S., Brunschwig B.S. (2021). X-ray photoelectron spectroscopy and resonant X-ray spectroscopy investigations of interactions between thin metal catalyst films and amorphous titanium dioxide photoelectrode protection layers. Chem. Mater..

[181] Navarro-Medina F., Oudijk A.E., Henriksen M.B., Garcia-Luis U., Gomez-San Juan A., Johansen T.A. (2024). Structural thermal optical performance (STOP) analysis and experimental verification of an hyperspectral imager for the HYPSO CubeSat. Opt. Lasers Eng..

[182] Wallace K., Collon M., Bavdaz M., Beijersbergen M., Fairbend R., Seguy J., Hoffmann M., Krumrey M. (2005). Development of micro-pore optics for x-ray applications. Optics for EUV, X-Ray, and Gamma-Ray Astronomy II.

[183] Yang X., Ling Z., Zhang C., Jin G., Zhang N., Zhang S.-N. (2023). Visible light transmittance of micro-pore optic plates for the wide-field x-ray telescope on the Einstein probe. Appl. Opt..

[184] Chen H., Zhang J., Falahati M., Weisse C. (2024). Optical system structural and thermal jitter analysis using Ansys Zemax OpticStudio. Free-Space Laser Communications XXXVI.

[185] Nab C.A.S. (2024). Einstein Probe Time Domain Astronomical Information Center. https://ep.bao.ac.cn/leia/cms/article/view?id=24&utm.

[186] Jin K., Tian Y., Erickson J.S., Puthoff J., Autumn K., Pesika N.S. (2012). Design and fabrication of gecko-inspired adhesives. Langmuir.

[187] Sun T., Liu Y., Tian Y. (2025). Bio-inspired controllable adhesion for robotics: Mechanisms, design, and future directions. Robotica.

[188] Sikdar S., Rahman M.H., Siddaiah A., Menezes P.L. (2022). Gecko-inspired adhesive mechanisms and adhesives for robots—A review. Robotics.

[189] Feder M., Giusti A., Vidoni R. (2022). An approach for automatic generation of the URDF file of modular robots from modules designed using SolidWorks. Procedia Comput. Sci..

[190] ASTM (2025). Standard Test Method for Peel or Stripping Strength of Adhesive Bonds. https://store.astm.org/d0903-98r17.html.

[191] Zhang G., Sun Y., Qian B., Gao H., Zuo D. (2020). Experimental study on mechanical performance of polydimethylsiloxane (PDMS) at various temperatures. Polym. Test..

[192] Anil Kumar V., Gupta R.K., Prasad M.J.N.V., Narayana Murty S.V.S. (2021). Recent advances in processing of titanium alloys and titanium aluminides for space applications: A review. J. Mater. Res..

[193] ASTM (2019). Standard Test Method for Apparent Shear Strength of Single-Lap-Joint Adhesively Bonded Metal Specimens by Tension Loading (Metal-to-Metal). https://store.astm.org/d1002-10r19.html?.

[194] Sameoto D., Khungura H., Benvidi F.H., Asad A., Liang T., Bacca M., Shyam V., Eggermont M., Hepp A.F. (2022). Chapter Fifteen-Space applications for gecko-inspired adhesives. Biomimicry for Aerospace.

[195] Chen T.G., Cauligi A., Suresh S.A., Pavone M., Cutkosky M.R., Testing Gecko-Inspired Adhesives With Astrobee Aboard the International Space Station: Readying the Technology for Space IEEE Robotics & Automation Magazine (Volume: 29, Issue: 3, September 2022). https://ieeexplore.ieee.org/document/9783137.

[196] Day P., Cutkosky M., McLaughlin A. (2012). Effects of gamma irradiation on adhesion of polymer microstructure-based dry adhesives. Nucl. Technol..

[197] Landau E.R. Gecko Grippers Moving on up; 2015/8/12 2015. https://www.jpl.nasa.gov/news/gecko-grippers-moving-on-up/.

[198] Lepora N.F., Verschure P., Prescott T.J. (2013). The state of the art in biomimetics. Bioinspiration Biomim..

[199] Ding H. (2022). Aerospace Mechatronics and Control Technology.

[200] Wanieck K., Hamann L., Bartz M., Uttich E., Hollermann M., Drack M., Beismann H. (2022). Biomimetics linked to classical product development: An interdisciplinary endeavor to develop a technical standard. Biomimetics.

[201] Messeri L. (2014). Earth as Analog: The Disciplinary Debate and Astronaut Training that Took Geology to the Moon. Astropolitics.

[202] Romo R., Andersen C., Edison K., Musilova M. (2021). Analog Field Sites on Hawai’i Island. Earth and Space 2021.

[203] Oguz E., Kubicek M., Clelland D. (2018). Failure modes and criticality analysis of the preliminary design phase of the Mars Desert Research Station considering human factors. Reliab. Eng. Syst. Saf..

[204] Zacny K., Cooper G. (2006). Considerations, constraints and strategies for drilling on Mars. Planet. Space Sci..

[205] Cermak A. (2017). Mars: Facts. https://science.nasa.gov/mars/facts/.

[206] Kahre M.A., Murphy J.R., Newman C.E., Wilson R.J., Cantor B.A., Lemmon M.T., Wolff M.J. (2017). The Mars dust cycle. Atmos. Clim. Mars.

[207] Diniega S., Bramson A.M., Buratti B., Buhler P., Burr D.M., Chojnacki M., Conway S.J., Dundas C.M., Hansen C.J., McEwen A.S. (2021). Modern Mars’ geomorphological activity, driven by wind, frost, and gravity. Geomorphology.

[208] Arrigo K. (2022). Research in analog environments to enable studies of ocean worlds. Oceanography.

[209] Preston L.J., Dartnell L.R. (2014). Planetary habitability: Lessons learned from terrestrial analogues. Int. J. Astrobiol..

[210] Anttila M., Ylikorpi T. Defining the Technical Requirements for Subsurface Mars Driller. Proceedings of the Sixth International Conference on Mars.

[211] Anttila M. (2005). Concept Evaluation of Mars Drilling and Sampling Instrument. Doctoral Thesis.

[212] ESA (2024). Einstein Probe Opens Its Wide Eyes to the X-Ray Sky. https://www.esa.int/Science_Exploration/Space_Science/Einstein_Probe_opens_its_wide_eyes_to_the_X-ray_sky.

[213] Zhang J., Qi L., Yang Y., Wang J., Liu Y., Cui W., Zhao D., Jia S., Li T., Chen T. (2022). Estimate of the background and sensitivity of the follow-up X-ray telescope onboard Einstein Probe. Astropart. Phys..

[214] Šimon V., Hudec R. (2022). Perspectives of the LOBSTER-EYE monitor in the soft X-ray observing the Galactic center region. J. High Energy Astrophys..

[215] Tamagawa T., Uchiyama K., Otsubo R., Yuasa T., Zhou Y., Mihara T., Ezoe Y., Numazawa M., Ishi D., Fukushima A. (2020). Multiplexing lobster-eye optics: A concept for wide-field X-ray monitoring. J. Astron. Telesc. Instrum. Syst..

[216] Qu L., Dai L. (2007). Gecko-foot-mimetic aligned single-walled carbon nanotube dry adhesives with unique electrical and thermal properties. Adv. Mater..

[217] Volpe R. JPL Robotics: Gecko-Like Adhesives for Orbital Debris Applications; 2025. https://www-robotics.jpl.nasa.gov/what-we-do/research-tasks/gecko-like-adhesives-for-orbital-debris-applications/.

[218] Jiang H., Hawkes E.W., Fuller C., Estrada M.A., Suresh S.A., Abcouwer N., Han A.K., Wang S., Ploch C.J., Parness A. (2017). A robotic device using gecko-inspired adhesives can grasp and manipulate large objects in microgravity. Sci. Robot..

[219] Pham V.A., Nguyen T.T., Lee B.R., Vo T.Q. (2020). Dynamic Analysis of a Robotic Fish Propelled by Flexible Folding Pectoral Fins. Robotica.

[220] Martínez-García E.A., Lavrenov R., Magid E. (2022). Robot fish caudal propulsive mechanisms: A mini-review. AI Comput. Sci. Robot. Technol..

[221] UNOOSA Space4SDGs: How Space can be Used in Support of the 2030 Agenda for Sustainable Development; 2025. https://www.unoosa.org/oosa/en/ourwork/space4sdgs/index.html.

[222] Saint-Sardos A., Aish A., Tchakarov N., Bourgoin T., Petit L.-M., Sun J.-S., Vignes-Lebbe R. (2024). Bioinspire-Explore: Taxonomy-Driven Exploration of Biodiversity Data for Bioinspired Innovation. Biomimetics.

[223] Emuna H., Borenstein N., Qian X., Kang H., Chan J., Kittur A., Shahaf D. Imitation of life: A search engine for biologically inspired design. Proceedings of the 38th AAAI Conference on Artificial Intelligence.

[224] Ruffridge B. Emulating nature: The future of sustainable design with BIDARA. Proceedings of the 34th Annual INCOSE International Symposium.

[225] Tchakarov N., Racca L., Peybernes T., Saint-Sardos A. (2023). A Scientific Corpus and Search Engine for Biomimetics. SSRN Electron. J..

[226] Biomimicry (2023). DesignLens: Life’s Principles. https://biomimicry.net/the-buzz/resources/designlens-lifes-principles/.

[227] Rangers M. (2022). Nature-based solutions public library by MIMICUS. http://www.lib.mimic.us/.

[228] Graeff E., Maranzana N., Aoussat A. (2021). Linkage, an Online Tool to Support Interdisciplinary Biomimetic Design Teams. J. Mech. Des..

[229] Mimicus (2021). Biomimicry. https://www.mimic.us/en.

[230] Hashemi Farzaneh H. (2020). Bio-inspired design: The impact of collaboration between engineers and biologists on analogical transfer and ideation. Res. Eng. Des..

[231] Qamar I.P., Stawarz K., Robinson S., Goguey A., Coutrix C., Roudaut A. Morphino: A nature-inspired tool for the design of shape-changing interfaces. Proceedings of the 2020 ACM Designing Interactive Systems Conference.

[232] Pusch R. (2019). Biomole. http://biomole.asknature.org/.

[233] Circulab (2018). Biomimicards: Embrace innovation inspired by nature. https://biomimicry.net/the-buzz/resources/biomimicry-gofish/.

[234] McInerney S.J., Khakipoor B., Garner A.M., Houette T., Unsworth C.K., Rupp A., Weiner N., Vincent J.F.V., Nagel J.K.S., Niewiarowski P.H. (2018). E2BMO: Facilitating User Interaction with a BioMimetic Ontology via Semantic Translation and Interface Design. Designs.

[235] Nagel J.K., Pittman P., Pidaparti R., Rose C., Beverly C. (2017). Teaching bioinspired design using C–K theory. Bioinspired Biomim. Nanobiomaterials.

[236] Biomimicry (2016). GoFish. https://biomimicry.net/the-buzz/resources/biomimicry-gofish/.

[237] Lenau T.A., Keshwani S., Chakrabarti A., Ahmed-Kristensen S. Biocards and level of abstraction. Proceedings of the 20th International Conference on Engineering Design (ICED 15) Vol 1: Design for life.

[238] Hashemi Farzaneh H., Helms K., Lindemann U. Visual representations as a bridge for engineers and biologists in bio-inspired design collaborations. Proceedings of the International Conference on Engineering Design (ICED 15) Vol 8: Innovation and Creativity.

[239] Biomimicry I. (2015). Nature’s Unifying Patterns. https://toolbox.biomimicry.org/core-concepts/natures-unifying-patterns/.

[240] Helms M., Goel A.K. (2014). The Four-Box Method: Problem Formulation and Analogy Evaluation in Biologically Inspired Design. J. Mech. Des..

[241] Deldin J.-M., Schuknecht M., Goel A.K., McAdams D.A., Stone R.B. (2013). The AskNature Database: Enabling Solutions in Biomimetic Design. Biologically Inspired Design: Computational Methods and Tools.

[242] Cheong H., Chiu I., Shu L.H., Stone R.B., McAdams D.A. (2011). Biologically Meaningful Keywords for Functional Terms of the Functional Basis. J. Mech. Des..

[243] Nagel J.K.S., Stone R.B., McAdams D.A. An Engineering-to-Biology Thesaurus for Engineering Design. Proceedings of the ASME 2010 International Design Engineering Technical Conferences and Computers and Information in Engineering Conference.

[244] Biomimicry (2008). AskNature. https://asknature.org/resource/biomimicry-taxonomy/.

[245] Vincent J.F., Bogatyreva O.A., Bogatyrev N.R., Bowyer A., Pahl A.-K. (2006). Biomimetics: Its practice and theory. J. R. Soc. Interface.

[246] Probe A., Oyake A., Chambers S.W., Deans M., Brat G., Cramer N., Roberts B., Hambuchen K. Space ROS: An open-source framework for space robotics and flight software. Proceedings of the AIAA SCITECH 2023 Forum.

[247] El Salibi O., Uyguroğlu M.K. Multi-Platforms Integration in Robotics: Simulating Robots Using CoppeliaSim, Choregraphe and Matlab. Proceedings of the 2024 9th International Conference on Robotics and Automation Engineering (ICRAE).

[248] Rivera Z.B., De Simone M.C., Guida D. (2019). Unmanned Ground Vehicle Modelling in Gazebo/ROS-Based Environments. Machines.

[249] Le Lidec Q., Jallet W., Montaut L., Laptev I., Schmid C., Carpentier J. (2024). Contact models in robotics: A comparative analysis. IEEE Trans. Robot..

[250] Yue H., Miao J., Zhang J., Fan C., Xu D. (2021). Simulation for senior undergraduate education of robot engineering based on Webots. Comput. Appl. Eng. Educ..

[251] Mower C., Stouraitis T., Moura J., Rauch C., Yan L., Behabadi N.Z., Gienger M., Vercauteren T., Bergeles C., Vijayakumar S. ROS-PyBullet Interface: A framework for reliable contact simulation and human-robot interaction. Proceedings of the 6th Conference on Robot Learning.

[252] Dosovitskiy A., Ros G., Codevilla F., Lopez A., Koltun V. CARLA: An open urban driving simulator. Proceedings of the 1st Annual Conference on Robot Learning.

[253] Tasora A., Serban R., Mazhar H., Pazouki A., Melanz D., Fleischmann J., Taylor M., Sugiyama H., Negrut D. (2016). Chrono: An open source multi-physics dynamics engine. High Performance Computing in Science and Engineering: Second International Conference, HPCSE 2015, Soláň, Czech Republic, May 25–28, 2015, Revised Selected Papers.

[254] Turco L., Zhao J., Xu Y., Tsourdos A. A study on co-simulation digital twin with MATLAB and AirSim for future advanced air mobility. Proceedings of the 2024 IEEE Aerospace Conference.

[255] Song Y., Naji S., Kaufmann E., Loquercio A., Scaramuzza D. Flightmare: A flexible quadrotor simulator. Proceedings of the 2020 Conference on Robot Learning.

[256] Nguyen K.D., Ha C. (2018). Development of Hardware-in-the-Loop Simulation Based on Gazebo and Pixhawk for Unmanned Aerial Vehicles. Int. J. Aeronaut. Space Sci..

[257] Pebrianti D., Suhaimi M.S., Bayuaji L., Hossain M.J. (2023). Exploring Micro Aerial Vehicle Mechanism and Controller Design Using Webots Simulation. Mekatronika: J. Intell. Manuf. Mechatron..

[258] Centelles D., Soriano A., Martí J.V., Marin R., Sanz P.J. UWSim-NET: An open-source framework for experimentation in communications for underwater robotics. Proceedings of the OCEANS 2019-Marseille.

[259] Manhães M.M.M., Scherer S.A., Voss M., Douat L.R., Rauschenbach T. UUV simulator: A gazebo-based package for underwater intervention and multi-robot simulation. Proceedings of the Oceans 2016 Mts/IEEE Monterey.

[260] Katara P., Khanna M., Nagar H., Panaiyappan A. Open source simulator for unmanned underwater vehicles using ros and unity3d. Proceedings of the 2019 IEEE Underwater Technology (UT).

[261] Paravisi M., Santos D.H., Jorge V., Heck G., Gonçalves L.M., Amory A. (2019). Unmanned surface vehicle simulator with realistic environmental disturbances. Sensors.

[262] Cieślak P. Stonefish: An advanced open-source simulation tool designed for marine robotics, with a ros interface. Proceedings of the OCEANS 2019-Marseille.

[263] Sacco E., Moon S.K. (2019). Additive manufacturing for space: Status and promises. Int. J. Adv. Manuf. Technol..

[264] Wang Y., Hao L., Li Y., Sun Q., Sun M., Huang Y., Li Z., Tang D., Wang Y., Xiao L. (2022). In-situ utilization of regolith resource and future exploration of additive manufacturing for lunar/martian habitats: A review. Appl. Clay Sci..

[265] Hammond M., Cichella V., Lamuta C. (2023). Bioinspired Soft Robotics: State of the Art, Challenges, and Future Directions. Curr. Robot. Rep..

[266] Zhang Y., Li P., Quan J., Li L., Zhang G., Zhou D. (2023). Progress, challenges, and prospects of soft robotics for space applications. Adv. Intell. Syst..

[267] Sanguino T.D.J.M. (2017). 50 years of rovers for planetary exploration: A retrospective review for future directions. Robot. Auton. Syst..

[268] Flores D., Sandhu S., White A., Yin A., Li A.L., Kang S., Wang Y., Chamorro L.P., Duduta M. (2025). RoboNautilus: A cephalopod-inspired soft robotic siphon for underwater propulsion. npj Robot..

[269] Gao C., Wang H., Zhao Y., Du X., Liu F. Design of a Novel Multi-Mode Deployable Metamorphic Aerospace Mechanism. Proceedings of the International Conference on Intelligent Robotics and Applications.

[270] Brambilla M., Ferrante E., Birattari M., Dorigo M. (2013). Swarm robotics: A review from the swarm engineering perspective. Swarm Intell..

[271] Sarker A., Ul Islam T., Islam M.R. (2025). A Review on Recent Trends of Bioinspired Soft Robotics: Actuators, Control Methods, Materials Selection, Sensors, Challenges, and Future Prospects. Adv. Intell. Syst..

